# Estimating dose—response relationships for vitamin D with coronary heart disease, stroke, and all-cause mortality: observational and revised Mendelian randomization analyses

**DOI:** 10.1016/S2213-8587(23)00287-5

**Published:** 2023-12-01

**Authors:** Emerging Risk Factors Collaboration/EPIC-CVD/Vitamin D Studies Collaboration, Eleni Sofianopoulou, Stephen K Kaptoge, Shoaib Afzal, Tao Jiang, Dipender Gill, Thomas E Gundersen, Thomas R Bolton, Elias Allara, Matthew G Arnold, Amy M Mason, Ryan Chung, Lisa AM Pennells, Fanchao Shi, Luanluan Sun, Peter Willeit, Nita G Forouhi, Claudia Langenberg, Stephen J Sharp, Salvatore Panico, Gunnar Engström, Olle Melander, Tammy YN Tong, Aurora Perez-Cornago, Margareta Norberg, Ingegerd Johansson, Verena Katzke, Bernard Srour, María José Sánchez, Daniel Redondo-Sánchez, Anja Olsen, Christina C Dahm, Kim Overvad, Magritt Brustad, Guri Skeie, Conchi Moreno-Iribas, N Charlotte Onland-Moret, Yvonne T van der Schouw, Konstantinos K Tsilidis, Alicia K Heath, Claudia Agnoli, Vittorio Krogh, Ian H de Boer, Camilla Jannie Kobylecki, Yunus Çolak, Armin Zittermann, Johan Sundström, Paul Welsh, Elisabete Weiderpass, Elom K Aglago, Pietro Ferrari, Robert Clarke, Marie-Christine Boutron, Gianluca Severi, Conor MacDonald, Rui Providencia, Giovanna Masala, Raul Zamora-Ros, Jolanda Boer, WM Monique Verschuren, Peggy Cawthon, Louise L Schierbeck, Cyrus Cooper, Matthias B Schulze, Manuela M Bergmann, Anke Hannemann, Stefan Kiechl, Hermann Brenner, Natasja M van Schoor, Juan R Albertorio, Carlotta Sacerdote, Allan Linneberg, Line L Kårhus, José María Huerta, Liher Imaz, Christel Joergensen, Yoav Ben-Shlomo, Annamari Lundqvist, John Gallacher, Naveed Sattar, Angela M Wood, Nicholas J Wareham, Børge G Nordestgaard, Emanuele Di Angelantonio, John Danesh, Adam S Butterworth, Stephen Burgess

**Affiliations:** 1 BHF Cardiovascular Epidemiology Unit, Department of Public Health and Primary Care, University of Cambridge, UK; 2 Copenhagen General Population Study, Department of Clinical Biochemistry, Herlev and Gentofte Hospital, Denmark; 3 Faculty of Health and Medical Sciences, Copenhagen University, Denmark; 4 Department of Epidemiology and Biostatistics, School of Public Health, Imperial College London, London, UK; 5 Vitas Ltd, Oslo, Norway; 6 NIHR Blood and Transplant Research Unit in Donor Health and Genomics, University of Cambridge, UK; 7 BHF Centre of Research Excellence, School of Clinical Medicine, Addenbrooke’s Hospital, University of Cambridge, UK; 8 Department of Medical Statistics, Informatics and Health Economics, Medical University of Innsbruck, Innsbruck, Austria; 9 Medical Research Council Epidemiology Unit, University of Cambridge, UK; 10 Berlin Institute of Health at Charité – Universitätsmedizin Berlin, Berlin, Germany; 11 Dipartimento Di Medicina Clinica E Chirurgia, Federico II University, Italy; 12 Department of Clinical Sciences Malmö, Lund University, Sweden; 13 Department of Emergency and Internal Medicine, Skåne University Hospital, Malmö, Sweden; 14 Cancer Epidemiology Unit, Nuffield Department of Population Health, University of Oxford, UK; 15 Department of Public Health and Clinical Medicine, Umeå University, Sweden; 16 Department of Odontology, Umeå University, Sweden; 17 Department of Cancer Epidemiology, German Cancer Research Center (DKFZ), Heidelberg, Germany; 18 EPIC Granada, Escuela Andaluza de Salud Pública (EASP), Granada, Spain; 19 Instituto de Investigación Biosanitaria ibs.GRANADA, Granada, Spain; 20 Centro de Investigación Biomédica en Red de Epidemiología y Salud Pública (CIBERESP), Madrid, Spain; 21 Department of Preventive Medicine and Public Health. University of Granada. Granada, Spain; 22 Danish Cancer Society Research Center, Copenhagen, Denmark; 23 Department of Public Health, Aarhus University, Denmark; 24 Department of Community Medicine, Faculty of Health Sciences, UiT the Arctic University of Norway, Norway; 25 The Public Dental Health Service Competence Centre of Northern Norway (TkNN), Tromsø, Norway; 26 Epidemiology, Prevention and Promotion Health Service, Public Health Institute of Navarra, Spain; 27 Julius Center for Health Sciences and Primary Care, University Medical Center Utrecht, Utrecht University, Netherlands; 28 Department of Epidemiology and Biostatistics, School of Public Health, Imperial College London, UK; 29 School of Medicine, University of Ioannina, Greece; 30 Epidemiology and Prevention Unit, Fondazione IRCCS Istituto Nazionale dei Tumori di Milano, Italy; 31 Department of Medicine, University of Washington, USA; 32 Clinic for Thoracic and Cardiovascular Surgery, Herz- und Diabeteszentrum Nordrhein-Westfalen, Bad Oeynhausen, Ruhr University Bochum, Germany; 33 Department of Medical Sciences, Uppsala University, Sweden; 34 BHF Glasgow Cardiovascular Research Centre, School of Cardiovascular and Metabolic Health, University of Glasgow, Glasgow, UK; 35 International Agency for Research on Cancer, France; 36 Clinical Trial Service Unit and Epidemiological Studies Unit, Nuffield Department of Population Health, Oxford, UK; 37 Université Paris-Saclay, UVSQ, Univ. Paris-Sud, Inserm U1018, Équipe "Exposome et Hérédité", CESP, Gustave Roussy, France; 38 Department of Statistics, Computer Science and Applications "G. Parenti" (DISIA), University of Florence, Italy; 39 Institute of Health Informatics Research, University College London, London, UK; 40 Institute for Cancer Research, Prevention and Clinical Network – ISPRO, Italy; 41 Unit of Nutrition and Cancer, Cancer Epidemiology Research Program, Catalan Institute of Oncology, Bellvitge Biomedical Research Institute (IDIBELL), Barcelona, Spain; 42 Centre for Nutrition and Health, National Institute for Public Health and the Environment (RIVM); 43 Research Institute, California Pacific Medical Center, USA; 44 Department of Epidemiology and Biostatistics, University of California, San Francisco, USA; 45 Cardiology Department, Nordsjælland University Hospital, Hillerød, Denmark; 46 Medical Research Council Lifecourse Epidemiology Unit, University of Southampton, Southampton, UK; 47 Faculty of Medicine, University of Southampton, Southampton, UK; 48 German Institute of Human Nutrition Potsdam-Rehbruecke, Nuthetal, Germany; 49 Institute of Nutritional Science, University of Potsdam, Germany; 50 Institute of Clinical Chemistry and Laboratory Medicine, DZHK (German Centre for Cardiovascular Research), Partner Site Greifswald, University Medicine Greifswald, Germany; 51 Department of Neurology, Medical University of Innsbruck, Innsbruck, Austria; 52 VASCage, Research Centre on Vascular Ageing and Stroke, Innsbruck, Austria; 53 Division of Clinical Epidemiology and Aging Research, German Cancer Research Center (DKFZ), Heidelberg, Germany; 54 Division of Preventive Oncology, German Cancer Research Center (DKFZ) and National Center for Tumor Diseases (NCT), Heidelberg, Germany; 55 German Cancer Consortium (DKTK), German Cancer Research Center (DKFZ), Heidelberg, Germany; 56 Network Aging Research, University of Heidelberg, Heidelberg, Germany; 57 Department of Epidemiology and Data Science, Amsterdam UMC, Vrije Universiteit Amsterdam, Amsterdam Public Health Research Institute, Netherlands; 58 Coalition to End Loneliness, USA; 59 Unit of Cancer Epidemiology, Città della Salute e della Scienza University-Hospital, Turin, Italy; 60 Center for Clinical Research and Prevention, Bispebjerg and Frederiksberg Hospital, The Capital Region, Denmark; 61 Department of Epidemiology, Murcia Regional Health Council, Instituto Murciano de Investigación Biosanitaria-Arrixaca, Murcia, Spain; 62 Public Health Division of Bizkaia, Ministry of Health of the Basque Government, Spain; 63 Biodonostia Health Research Institute, Donostia-San Sebastian, Spain; 64 Steno Diabetes Center, Copenhagen, Denmark; 65 Population Health Sciences, University of Bristol, UK; 66 Finnish Institute for Health and Welfare, Helsinki, Finland; 67 Department of Psychiatry, University of Oxford, Oxford, UK; 68 Health Data Research UK Cambridge, Wellcome Genome Campus and University of Cambridge, UK; 69 The Alan Turing Institute, UK; 70 The Copenhagen City Heart Study, Frederiksberg Hospital, Copenhagen University Hospital; 71 Department of Human Genetics, Wellcome Sanger Institute, Hinxton, UK; 72 Medical Research Council Biostatistics Unit, University of Cambridge, UK

**Keywords:** 25-hydroxyvitamin D, Vitamin D deficiency, Mendelian randomization, cardiovascular disease, all-cause mortality

## Abstract

**Background:**

Randomized trials of vitamin D supplementation for cardiovascular disease and all-cause mortality have generally reported null findings. However, generalizability of results to individuals with low vitamin D status is unclear. We characterized dose—response relationships between 25-hydroxyvitamin D [25(OH)D] concentrations and risk of coronary heart disease (CHD), stroke, and all-cause mortality in observational and Mendelian randomization frameworks.

**Methods:**

Observational analyses were conducted in 33 prospective studies comprising 500,962 individuals with no known history of CHD or stroke at baseline. Mendelian randomization analyses were conducted in four population-based cohort studies comprising 386,406 middle-aged individuals of European ancestries, including 33,546 CHD cases, 18,166 stroke cases, and 27,885 deaths.

**Findings:**

Observational analyses suggested threshold relationships for incident CHD, stroke, and mortality outcomes. In population-wide genetic analyses, there were no associations of genetically-predicted 25(OH)D with CHD (odds ratio [OR] per 10 nmol/L higher genetically-predicted 25(OH)D concentration 0·98, 95% confidence interval [CI] 0·95-1·01), stroke (OR 1·01, 95% CI 0·97-1·05), or all-cause mortality (OR 0·99, 95% CI 0·95-1·02). Null findings were also observed in genetic analyses for cause-specific mortality outcomes, and in stratified genetic analyses for all outcomes at all observed levels of 25(OH)D concentrations.

**Interpretation:**

Stratified Mendelian randomization analyses suggest a lack of causal relationship for 25(OH)D concentrations with both cardiovascular and mortality outcomes for individuals at all levels of 25(OH)D. Our findings suggest that substantial reductions in mortality and cardiovascular morbidity due to long-term low-dose vitamin D supplementation are unlikely even if targeted at individuals with low vitamin D status.

**Funding:**

British Heart Foundation, Medical Research Council, National Institute for Health Research, Health Data Research UK, International Agency for Research on Cancer.

## Introduction

Vitamin D is an essential nutrient obtained from sunlight, dietary intake, and supplementation.^1^ Observational epidemiological studies have consistently found that low concentrations of circulating 25-hydroxyvitamin D [25(OH)D] (a metabolite used as a clinical indicator of vitamin D status) are associated with higher risk of cardiovascular disease (CVD) and all-cause mortality, as well as other chronic diseases.^2,3^ However, several large randomized trials of vitamin D supplementation have reported null results,^4–6^ casting doubt on the observational evidence. However, as trials have typically recruited participants irrespective of baseline 25(OH)D concentration, they have had limited power to test supplementation effects among subgroups with low 25(OH)D concentrations.^7^

An efficient approach for assessing the potential causal effect of vitamin D supplementation is Mendelian randomization. Mendelian randomization uses genetic variants specifically related to a particular exposure to compare genetically-defined population subgroups with different average levels of the exposure. The independent segregation of alleles at conception means that these genetically-defined subgroups should not differ systematically with respect to confounding variables, creating a natural experiment analogous to a randomized trial.^8^ Therefore, compared with conventional observational analyses, Mendelian randomization analyses can provide more reliable insights into causal relationships between risk factors and disease outcomes. Previous Mendelian randomization analyses have reported null associations of genetically-predicted 25(OH)D concentrations with coronary heart disease (CHD)^9,10^ and ischaemic stroke.^11,12^ An inverse association has been observed between genetically-predicted 25(OH)D and all-cause mortality.^13^ Null findings have been observed for several further outcomes, including other cardiovascular diseases and cancers.^14^

Most previous Mendelian randomization analyses assumed a linear dose—response relationship between genetically-predicted 25(OH)D and CVD. However, some observational analyses have reported non-linear associations,^15,16^ suggesting methods that assume linearity may not provide an accurate picture of the dose—response relationship. Several non-linear Mendelian randomization analyses have been performed (see Supplementary Table 1 for list). However, such analyses can be severely biased if genetic effects on the exposure vary in the population^17^. This manuscript is a revised version of a previous publication that was affected by this bias^18^.

Here, we conduct the largest observational analysis to date to characterize the shape of association between 25(OH)D concentrations and CVD outcomes in an individual-participant data meta-analysis of 33 prospective studies. We then perform stratified Mendelian randomization analyses using the doubly-ranked method^19^, which is more robust to variation in the genetic effects on the exposure, to assess evidence for potential causal effects of 25(OH)D concentrations on risk of major CVD outcomes, all-cause mortality, and cause-specific mortality for population subgroups with different average 25(OH)D concentrations.

## Methods

### Study populations

Observational analyses were conducted in UK Biobank, the European Prospective Investigation into Cancer and Nutrition Cardiovascular Disease study (EPIC-CVD), and 31 studies from the Vitamin D Studies Collaboration (VitDSC). Genetic analyses were conducted in UK Biobank, EPIC-CVD, and two Danish population-based studies. Only baseline measurements of 25(OH)D were used in analyses.

UK Biobank is a prospective cohort study of around 500,000 people aged 40 to 69 years at baseline, recruited in 2006-2010 from the United Kingdom and followed-up for a median of 10·9 years.^20^ For observational analyses, we analysed 384,721 individuals without previous known CVD at baseline. For genetic analyses, we included data on 333,002 unrelated individuals of European ancestries. EPIC-CVD is a case-cohort study derived from a cohort of over 500,000 individuals recruited at 23 centres across 10 European countries.^21,22^ Participants were followed-up for a median of 9·5 years. We analysed 26,336 individuals without previous known CVD at baseline in observational analyses, and 22,142 individuals of European ancestries in genetic analyses. VitDSC comprises 89,915 participants from 31 mostly population-based, prospective studies across 11 countries. Individual-participant data on 25(OH)D concentrations, conventional cardiovascular risk factors, and major incident cardiovascular morbidity and mortality were available for 67,992 individuals without previous known CVD. The Copenhagen City Heart Study (CCHS) and Copenhagen General Population Study (CGPS) are prospective cohort studies in the Danish population.^23,24^ CCHS was initiated in 1976 and participants were followed up periodically until 2018. Median follow-up was 28·6 years. CGPS was initiated in 2003 and has a median follow-up of 8·8 years. For genetic analyses, we analysed 31,262 individuals from both studies with genetic data and a 25(OH)D measurement. For all studies, written informed consent was obtained from participants and approval was obtained from relevant ethics committees.

### Vitamin D measurement and classification

Concentrations of 25(OH)D in blood were measured using the DiaSorin Liaison immunoassay analyser (UK Biobank and Copenhagen studies) or liquid chromatography-tandem mass spectrometry (EPIC-CVD). In VitDSC, concentrations were measured by radioimmunoassay, direct chromatographic approaches, or other immunoassays (Supplementary Table 2). Measurements were seasonally adjusted in each study to correspond to a measurement taken in autumn by subtracting the study-specific mean 25(OH)D concentration for the season the measurement was taken in and then adding the study-specific mean 25(OH)D concentration for autumn measurements. In EPIC-CVD, centre-specific means were used rather than study-specific means.

### Outcomes

Outcomes were ascertained using International Classification of Diseases, Tenth Revision (ICD-10) codes. Primary outcomes were CHD, defined as fatal ischaemic heart disease (ICD-10 code: I20-I25) or non-fatal myocardial infarction (I21-I23); stroke, defined as any cerebrovascular disease (I60-I69); and all-cause mortality. We performed secondary analyses for cause-specific mortality divided into cardiovascular mortality, cancer mortality, or non-cardiovascular non-cancer mortality using ICD-10 codes (Supplementary Table 3). Observational analyses included incident events only. Supplementary genetic analyses were performed restricting to incident CHD and stroke events, and separating ischaemic stroke (I63-I64) and haemorrhagic stroke (I60-I61).

### Genetic variants

To minimize potential bias due to horizontal pleiotropy, we considered genetic variants from four gene regions previously shown to be strongly associated with 25(OH)D and implicated in the transport, metabolism, and synthesis of vitamin D^25^ – *GC, DHCR7, CYP2R1*, and *CYP24A1*. The *GC* gene encodes vitamin D binding protein. The *DHCR7* gene product converts 7-dehydrocholesterol to cholesterol, reducing 7-dehydrocholesterol available for conversion to previtamin D_3_ by solar radiation. The *CYP2R1* gene encodes vitamin D 25-hydroxylase, a regulator of 25(OH)D synthesis through 25-hydroxylation of vitamin D in the liver. The *CYP24A1* gene product inactivates the active form of vitamin D (1α25(OH)_2_D). To maximize the variance explained by the genetic instrument, we considered available variants at each genetic locus and selected variants associated with 25(OH)D concentrations using a stepwise selection method (Supplementary Methods). For UK Biobank and EPIC-CVD, 21 variants were included in the analysis (Supplementary Table 4). For the Copenhagen studies, due to limited availability, analyses were restricted to three variants: two from the *CYP2R1* locus (rs12794714 and rs117913124) and one from the *DHCR7* locus (rs7944926).

We also considered a score based on 71 genetic variants from across the genome (“genome-wide score”) previously demonstrated to be associated with 25(OH)D concentrations at a genome-wide level of statistical significance^26^.

### Statistical methods

#### Observational analysis

Observational associations were assessed by inverse-variance weighted random-effects meta-analysis of study-specific hazard ratios (HRs), calculated using Cox proportional hazards regression models stratified by sex and (where appropriate) centre or trial arm. Principal analyses were adjusted for age at blood draw for 25(OH)D measurement, calendar month of blood draw, smoking status (current versus other), total cholesterol, high-density lipoprotein (HDL) cholesterol, systolic blood pressure, known history of diabetes, and body mass index; all measured at study baseline.

The primary dose—response analyses assessed the shape of association between 25(OH)D and outcomes by meta-analysis of fractional polynomials adjusted for the conventional risk factors. Supplementary analyses combined study-specific hazard ratios by tenths of 25(OH)D and plotted the pooled hazard ratios against the pooled mean 25(OH)D within each tenth.

#### Genetic analysis

We constructed a genetic risk score (GRS) weighted by the conditional associations of the genetic variants with 25(OH)D concentration in UK Biobank (Supplementary Table 4). Mendelian randomization estimates were calculated using the ratio method by dividing the genetic association with the outcome by the genetic association with 25(OH)D concentration, and scaling the estimate to a 10 nmol/L difference in genetically-predicted 25(OH)D concentration. Genetic associations were estimated using logistic regression for disease outcomes, and using linear regression for 25(OH)D concentrations. All regression models were adjusted for age at baseline, sex, centre (for UK Biobank and EPIC-CVD), and ten genetic principal components of ancestry. We assessed specificity of the GRS by testing its associations with a range of cardiovascular risk factors in the UK Biobank study.

In addition to analyses conducted in the overall study sample to estimate population-averaged causal effects, we also conducted stratified analyses in strata of the population constructed using the doubly-ranked stratification method^19^, a statistical method for constructing strata of the population with different average levels of the exposure such that stratum membership is independent of the genetic instrument. Stratification on 25(OH)D levels directly would induce collider bias, meaning that the distribution of the genetic instrument would vary between strata, and the instrumental variable assumptions would be violated in the strata. A previously-proposed method that stratifies on residual values of 25(OH)D (that is, the non-genetic component of 25(OH)D) requires the genetic effect on 25(OH)D concentrations to be linear and homogeneous in the population to provide unbiased estimates; an assumption that is generally implausible and is violated in this case^17^. We calculated Mendelian randomization estimates for 10 equally-sized strata using the ratio method with the GRS as an instrumental variable, and combined stratum-specific estimates across studies using fixed-effect meta-analysis. All estimates from the doubly-ranked method were averaged across 100 iterations of the method (see Supplementary Material for details).

All statistical analyses were performed in R version 4·0·5 (R Foundation for Statistical Computing, Vienna, Austria), Stata/SE 15·1 (College Station, TX, USA), or BOLT-LMM version 2·3·4 (Broad Institute, MS, USA).

### Role of the funding source

The funders of the study had no role in study design, data collection, data analysis, data interpretation, or writing of the report. The corresponding author had full access to all the data in the study and had final responsibility for the decision to submit for publication.

## Results

Table 1 shows baseline characteristics of 386,406 participants in the four studies included in genetic analyses (details of 500,962 participants included in observational analyses are in Supplementary Tables 5 and 6). Mean age of participants ranged from 54·8 to 57·5 years, with similar numbers of men and women in each study. Mean season-shifted 25(OH)D concentrations (corresponding to an autumn measurement) were 54·5 nmol/L in UK Biobank, 46·9 nmol/L in EPIC-CVD, and 53·8 nmol/L in the Copenhagen studies (Supplementary Table 7). Mean 25(OH)D estimates did not notably differ by assay type (Supplementary Figure 1). The focused GRS explained 4·7% of the variance in 25(OH)D concentrations in UK Biobank, 5·8% in EPIC-CVD, and 1·8% in the Copenhagen studies. This GRS was not associated with a range of cardiovascular risk factors in UK Biobank, with the exception of body mass index and HDL-cholesterol, although these associations were small in magnitude (Supplementary Figure 2). The genome-wide score was strongly associated with LDL-cholesterol and triglycerides (Supplementary Figure 3), and so Mendelian randomization estimates using this score are unreliable.

**Figure 1:**
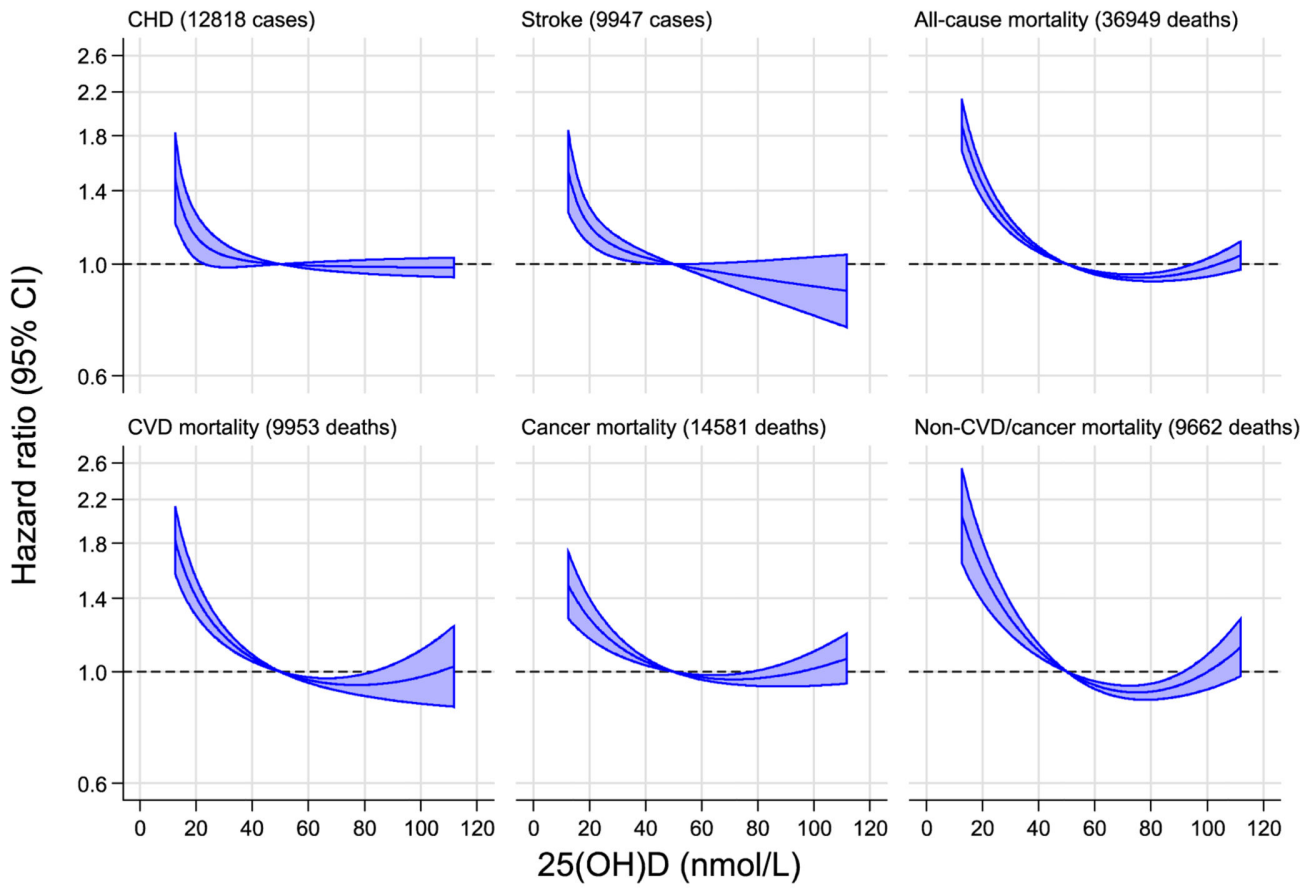
Observational associations of 25(OH)D concentration with outcomes. Reference value is 50 nmol/L, which is close to the mean concentration of 25(OH)D in most contributing studies. The shaded area represents the 95% confidence interval for the dose—response curve. Study-specific analyses involved fractional polynomial modelling of continuous associations of 25(OH)D and outcomes using Cox regression stratified by sex, and (where appropriate) trial arm and centre, and adjusted for conventional cardiovascular risk factors, namely: age at blood draw for 25(OH)D measurement, calendar month of blood draw, smoking status (current versus other), total cholesterol, high-density lipoprotein (HDL) cholesterol, systolic blood pressure, known history of diabetes, and body-mass index (all measured at baseline), followed by random effects meta-analysis (see Methods).

Observational associations had a similar non-linear shape for all outcomes (Figure 1 and Supplementary Figure 4): at low concentrations of 25(OH)D, there was an inverse association; whereas at higher concentrations of 25(OH)D, the association was null (for CVD outcomes) or weakly positive (for mortality outcomes). For CHD and stroke, there was no strong association for 25(OH)D concentrations above 50 nmol/L, but a progressively steeper association was observed below this threshold. For all-cause mortality, the strength of the inverse association at lower 25(OH)D concentrations was stronger and began at a higher 25(OH)D concentration.

The shapes of the observational associations in the three primary data sources were broadly similar (Supplementary Figure 5). Dose—response findings were also similar in supplementary analyses that combined study-specific hazard ratios by deciles of 25(OH)D or according to the four 25(OH)D categories (Supplementary Figure 6 and Supplementary Table 8).

**Figure 2:**
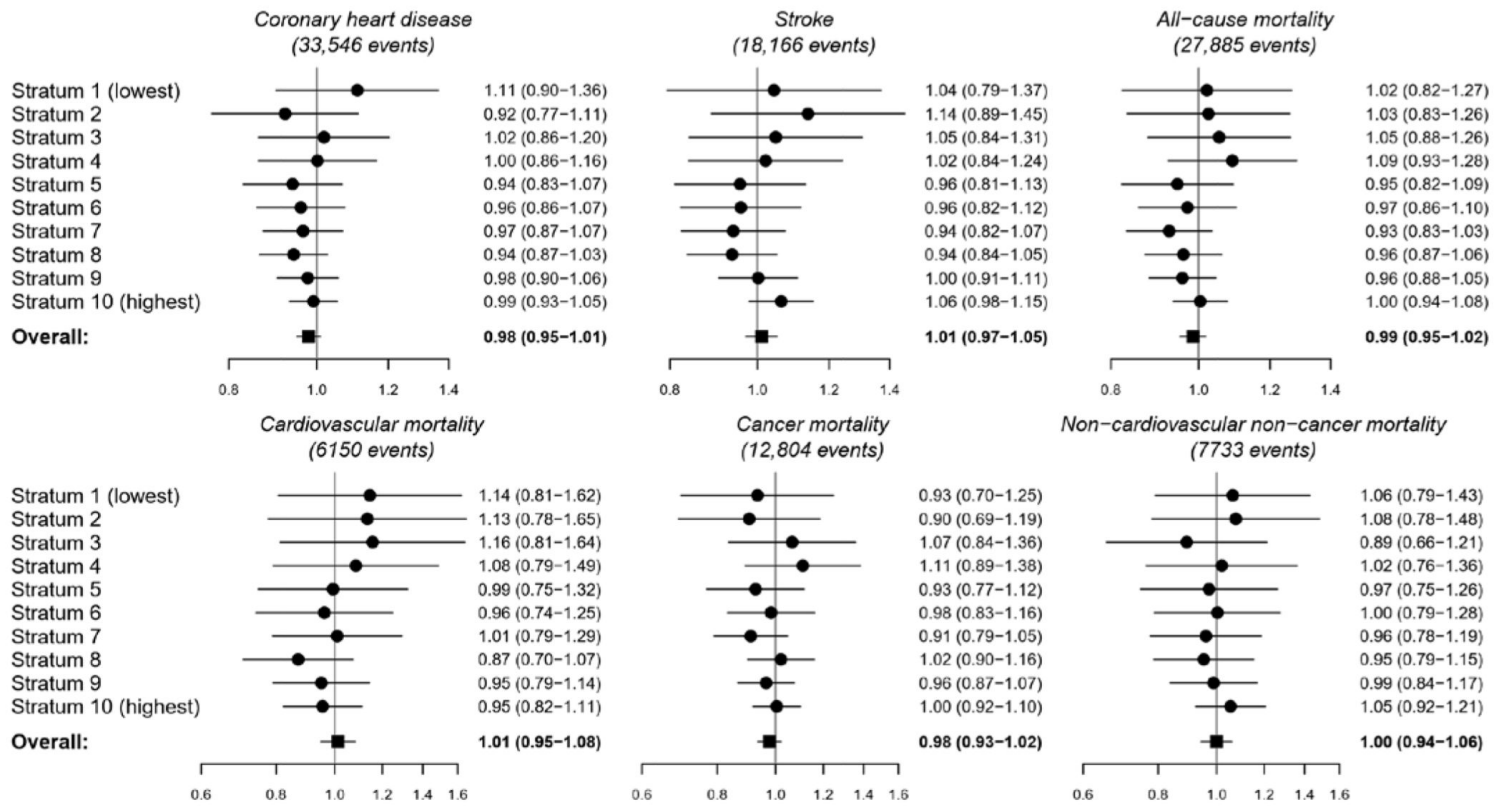
Mendelian randomization estimates in overall population and strata of residual 25(OH)D concentrations. Estimates (95% confidence intervals) represent odds ratios per 10 nmol/L higher genetically-predicted concentration of 25(OH)D in strata of the population defined by residual concentration of 25(OH)D.

Mendelian randomization estimates, representing the odds ratio (OR) per 10 nmol/L higher genetically-predicted 25(OH)D concentration, are presented in Figure 2 (top row) for primary outcomes (study-specific estimates in Supplementary Table 9). In overall analyses (that is, population-averaged estimates across the full range of the 25(OH)D concentration distribution), there was no association between genetically-predicted 25(OH)D and CHD (OR 0·98, 95% confidence interval [CI] 0·95-1·01, p=0·18), stroke (OR 1·01, 95% CI 0·97-1·05, p=0·61), or all-cause mortality (OR 0·99, 95% CI 0·95-1.02, p=0·39). However, there was some evidence of an overall inverse association with all-cause mortality in the Copenhagen studies (OR 0·89, 95% CI 0·80-0·99, p=0·030). In stratified analyses (that is, stratum-averaged estimates for strata of the population with different average 25(OH)D concentrations), there were no clear associations between genetically-predicted 25(OH)D and the main outcomes in any stratum, and no discernible trends in the stratum-specific estimates. The distributions of 25(OH)D concentrations in strata are provided in Table 2.

Similar results were observed for supplementary analyses that considered incident stroke outcomes only, ischaemic stroke only (Supplementary Table 10), and incident CHD outcomes only (Supplementary Table 11). Estimates using the pleiotropic genome-wide score are presented in Supplementary Table 12. The precision of estimates varied between strata; this is because genetic associations with 25(OH)D were much stronger in individuals with high 25(OH)D concentrations than in individuals with low 25(OH)D concentrations (Supplementary Figure 7). In UK Biobank, genetic associations with 25(OH)D were 5.0 times stronger in the highest decile compared with the lowest decile; in EPIC-CVD, this ratio was 4.6, and in the Copenhagen studies, this ratio was 9·7.

Mendelian randomization estimates for cause-specific mortality in UK Biobank and the Copenhagen studies are presented in Figure 2 (bottom row; study-specific estimates in Supplementary Table 13). In overall analyses, there was no association between genetically-predicted 25(OH)D and cardiovascular mortality (OR 1·01, 95% CI 0·95-1·08, p=0·71), cancer mortality (OR 0·98, 95% CI 0·93-1·02, p=0·29), or non-cardiovascular non-cancer mortality (OR 1·00, 95% CI 0·94-1.06, p=0·99). Similarly, null findings were obtained for mortality outcomes in all stratum-specific analyses.

**Figure 3:**
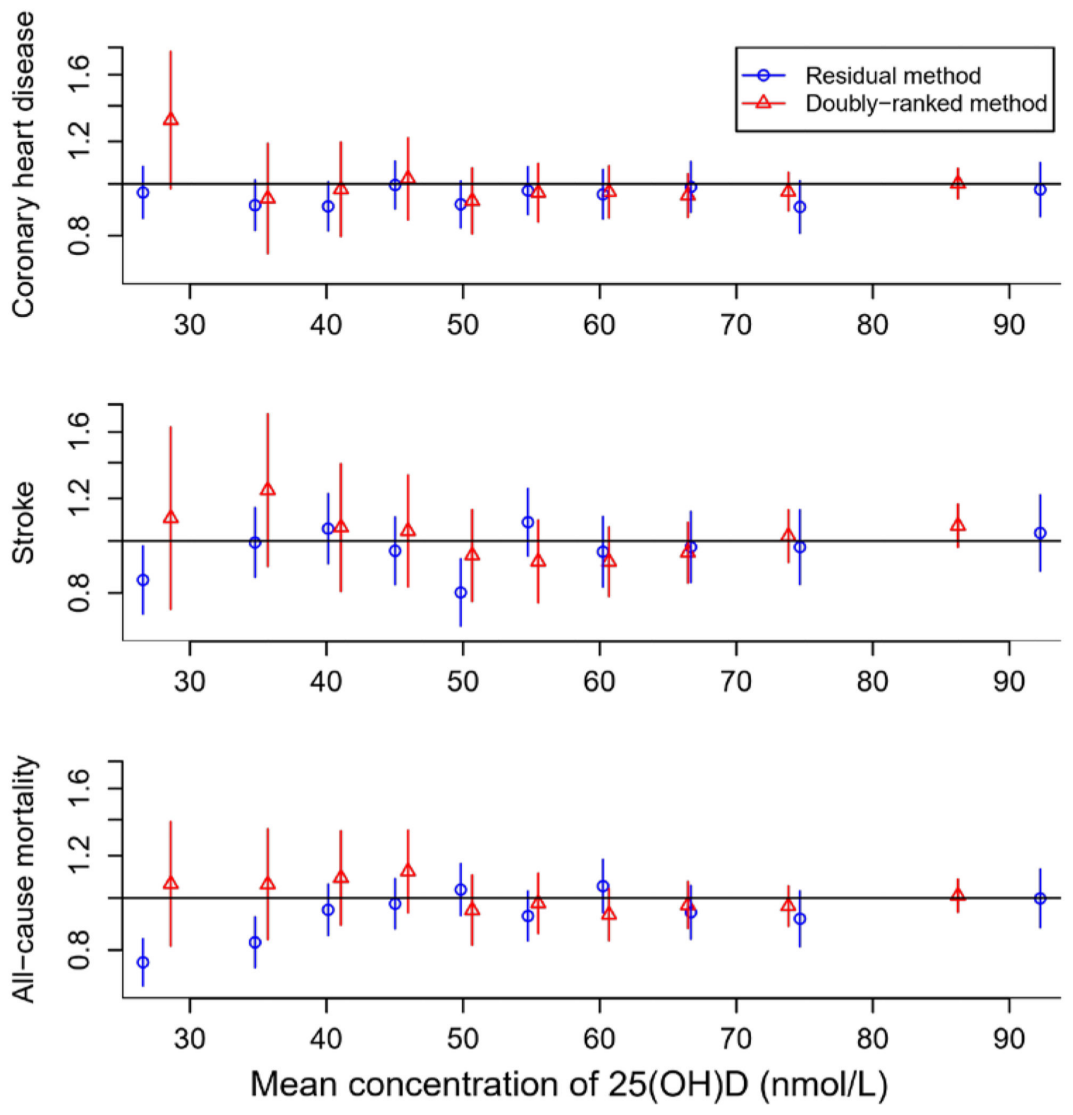
Stratified Mendelian randomization estimates for primary outcomes in UK Biobank from residual and doubly-ranked methods. Estimates (95% confidence intervals) represent odds ratios per 10 nmol/L higher genetically-predicted concentration of 25(OH)D in strata of the population defined by residual concentration of 25(OH)D. Estimates from the residual method (unreliable when the genetic effect on the exposure vary in the population) are shown as blue triangles. Estimates from the doubly-ranked method (more reliable when the genetic effect on the exposure vary in the population) are shown as red triangles.

A comparison between estimates from the doubly-ranked method and the residual method for primary outcomes in the UK Biobank is presented in Figure 3 (Supplementary Figure 8 for mortality outcomes). Estimates differ substantially at low levels of 25(OH)D, with the residual method giving estimates in the protective direction, with confidence intervals that exclude the null for stroke and all-cause mortality, and the doubly-ranked method giving estimates in the harmful direction for primary outcomes, although compatible with the null in all cases.

## Discussion

In observational analyses, we found evidence for non-linear dose—response relationships of 25(OH)D concentrations with CVD and all-cause mortality. However, population-averaged estimates from our genetic analyses suggest that interventions to increase 25(OH)D concentrations are unlikely to translate into substantial risk reductions for CVD or all-cause mortality in the population overall. Similarly, genetic analyses conducted in individuals with low 25(OH)D concentrations provided no evidence supporting a causal relationship between 25(OH)D concentrations and cardiovascular or mortality outcomes. Our results suggest that substantial reductions in mortality and cardiovascular morbidity due to long-term low-dose vitamin D supplementation are unlikely even from trials targeted at individuals with low vitamin D status.

The majority of large prior trials for CVD and mortality were conducted in broadly-selected groups of the population, so had limited reliability to assess evidence for causality in individuals with low 25(OH)D concentrations. Most previous Mendelian randomization analyses did not consider estimates for strata of the population defined according to baseline 25(OH)D concentrations, and hence have not considered the shape of the causal relationship between 25(OH)D concentrations and CVD or all-cause mortality. Previous non-linear Mendelian randomization investigations conducted by ourselves^18^ and by others^27^ used the residual stratification method, which assumes that the effect of the genetic instrument on the exposure is linear and homogeneous in the population. These analyses suggested a protective causal effect of 25(OH)D at low 25(OH)D concentrations, as has been suggested for respiratory tract infections^28^. However, the residual method cannot reliably detect when the constant genetic effect assumption is violated^19^, and when it is violated, the method can produce biased results that reflect observational confounded associations rather than causal relationships^17^.

We have several reasons for greater trust in the updated null results from the doubly-ranked method over the previous results from the residual stratification method. First, genetic associations with the exposure varied strongly between strata, violating the assumption required by the residual method. There are several plausible reasons why genetic associations with 25(OH)D may be smaller in individuals with lower 25(OH)D concentrations: if genetic variants act via biological mechanisms relating to 25(OH)D synthesis or metabolism, then we may expect smaller magnitudes of effect in individuals with low levels of 25(OH)D synthesis and metabolism. Genetic variants may have similar proportional effects on 25(OH)D concentrations, rather than constant additive effects. Secondly, in the UK Biobank dataset, significant associations with confounders have been observed in 25(OH)D strata defined by the residual method^17^. This provides empirical evidence that the Mendelian randomization assumptions are violated in strata defined by the residual method, even if they are not violated in the overall population. Thirdly, a sensitivity analysis for the residual method log-transforming 25(OH)D concentrations before stratification provided substantially attenuated estimates in low 25(OH)D strata^17^. Log-transformation reduces the difference between genetic associations with 25(OH)D at the top and bottom of the distribution of 25(OH)D concentrations, and so should reduce bias due to violation of the constant genetic effect assumption. Fourthly, theoretical investigations have shown greater bias in estimates from the residual method compared to the doubly-ranked method in a wide range of simulated scenarios when the constant genetic effect assumption does not hold^17,19^. Finally, there were discrepancies in results from the residual method in our previous analysis; namely, the overall estimate for cardiovascular mortality was positive (but non-significant), even though all stratum-specific estimates were negative^29^. While this is possible for observational associations (it is known as “Simpson’s paradox”), this is a logical impossibility if all estimates have a causal interpretation. Hence, we have strong empirical and methodological reasons to doubt our previously published finding supporting a protective effect of 25(OH)D in low vitamin D status individuals^18^. However, all statistical methods, particularly those for inferring causal relationships, make untestable assumptions, which cannot be empirically verified. Hence, even for the doubly-ranked method, there remains intrinsic uncertainty in the validity of results: in the validity of the genetic variants as instrumental variables, in the assumptions required for non-linear Mendelian randomization, and so on.

Our revised investigation has several strengths. The Mendelian randomization design means that estimates are less susceptible to bias from confounding and reverse causation than those from conventional observational analyses. Our focused genetic instrument for vitamin D afforded strong statistical power and biological specificity, minimizing the potential for bias due to horizontal pleiotropy arising from use of variants that do not have specific effects on vitamin D pathways. The focused score was not associated with major cardiovascular risk factors, providing empirical evidence to support the Mendelian randomization assumptions. While the genetic instrument only explained a limited proportion of the variance in 25(OH)D levels, vitamin D supplementation in a randomized controlled trial would only explain a limited proportion of the variance in 25(OH)D levels. In the VITAL trial, supplementation only explained 26% of the variance in 25(OH)D levels^4^. While the limited proportion of variance explained affects the power to detect a causal effect, it does not preclude causal inferences either in a trial or a Mendelian randomization investigation.

However, there are also potential limitations. Firstly, the Mendelian randomization assumptions state that the only causal pathway from the genetic variants to the outcome is via 25(OH)D concentrations. While our variants are all from gene regions specifically relevant to vitamin D biology, variants in the *CYP24A1* gene region are known to associate with circulating calcium levels. Secondly, while weaker than the assumptions required by the residual stratification method, non-linear Mendelian randomization analyses using the doubly-ranked method require additional assumptions beyond standard population-based Mendelian randomization analyses; namely the “rank preserving assumption” that the ranking of participants by their exposure values would be the same for all values of the genetic instrument. This assumption is generally plausible, but cannot be empirically tested. Thirdly, to reduce the scope for confounding by ethnicity (“population stratification”), our analyses were limited to middle-aged participants of European ancestries. This means that our findings may not be applicable to other populations. In particular, further analyses are needed to assess the potential effect of vitamin D supplementation in individuals with dark skin, as this correlates with lower 25(OH)D concentrations. Fourthly, UK Biobank and EPIC-CVD are not fully representative samples of the UK and European populations, further limiting the applicability of findings. Selection into these studies is dependent on age, sex, and other covariates, which can lead to bias in Mendelian randomization estimates^30^. While the effect of moderate selection bias on Mendelian randomization estimates is often slight, one specific concern in non-linear Mendelian randomization is that selection bias may affect stratum-specific estimates non-differentially; that is, bias may be greater in some strata than in others. Fifthly, fewer genetic variants were available in the Copenhagen studies, limiting comparability between datasets. However, while this will reduce power for analysis, it should not lead to bias. Sixthly, we do not have information from all studies on the accuracy of 25(OH)D measurements from external quality control programs. However, there was no indication that mean 25(OH)D estimates varied by assay type. As any such variation is likely non-differential to morbidity and mortality outcomes, it would bias the results toward the null. Seventhly, our primary genetic analyses for CVD considered both prevalent and incident events. Stratification into categories according to residual 25(OH)D concentration may therefore be affected by reverse causation. However, genetic associations with disease outcomes within each of the strata will not be affected by reverse causation, as genotype is fixed from conception. Finally, while our Mendelian randomization analyses provided confidence intervals that overlapped the null in all strata, it is not possible to prove a null finding; we may not have sufficient power to detect a small causal effect, particularly in the stratified analyses, as the sample size is naturally reduced for these analyses.

In conclusion, while observational analyses found a threshold association between 25(OH)D and cardiovascular and mortality outcomes, this was not confirmed in genetic analyses. This suggests the absence of a causal effect of 25(OH)D on cardiovascular and mortality outcomes, even at low 25(OH)D concentrations.

## Supplementary Material

Supplementary material

## Figures and Tables

**Table 1:** Characteristics of participants in the genetic analyses by study

	UK Biobank	EPIC-CVD	Copenhagen studies
**Participants**	333,002	22,142	31,262
**Age at baseline**	57·1 ± 8·1	54·8 ± 9·4	57·5 ± 12·9
**Sex**			
Female	177,733 (53·4)	11,426 (51·6)	17,311 (55·4)
Male	155,269 (46·6)	10,716 (48·4)	13,951 (44·6)
**25(OH)D concentration (nmol/L)**	54·5 ± 19·6	46·9 ± 16·4	53·8 ± 25·9
**CHD events**	22,363 (6·7)	5942 (26·8)	5241 (16·8)
**Stroke events**	10,489 (3·1)	5478 (24·7)	2199 (7·0)
**Deaths**	20,340 (6·1)	- ^1^	7545 (24·1)
**BMI (kg/m^2^)**	27·3 ± 4·8	26·7 ± 4·3	25·9 ± 4·2
**SBP (mmHg)**	137·5 ± 18·6	137·4 ± 21·3	140·1 ± 21·0
**Smoking**			
Current	34,085 (10·2)	6867 (31·0)	8387 (26·9)
Other	298,940 (89·8)	15,275 (69·0)	22,784 (73·1)
**Diabetes**			
Known history	15,822 (4·8)	1234 (5·6)	1244 (4·0)
No known history	317,203 (95·2)	20,908 (94·4)	30,018 (96·0)

Values represent mean ± standard deviation for continuous traits or N (%) for categorical traits. 25(OH)D concentrations are season-shifted to correspond to a measurement taken in autumn. Abbreviations: 25(OH)D = 25-hydroxyvitamin D, BMI = body mass index, CHD = coronary heart disease, SBP = systolic blood pressure.

1 EPIC-CVD was specifically designed as a case-cohort study of cardiovascular disease outcomes, and therefore does not contribute to the analysis of non-cardiovascular disease or all-cause mortality outcomes.

**Table 2:** Distribution of 25(OH)D concentrations in strata by study

	UK Biobank	EPIC-CVD	Copenhagen studies
Stratum 1 (lowest)	28.6 (22.7, 38.0)	25.3 (19.5, 34.2)	25.3 (18.3, 36.2)
Stratum 2	35.7 (27.0, 45.0)	31.9 (24.1, 40.1)	33.6 (23.1, 44.8)
Stratum 3	41.1 (31.9, 51.0)	36.4 (28.5, 45.1)	40.1 (29.1, 51.7)
Stratum 4	46.0 (36.1, 56.5)	40.3 (32.2, 49.3)	46.0 (34.7, 57.9)
Stratum 5	50.7 (40.2, 61.8)	44.1 (35.7, 53.4)	51.6 (39.9, 64.1)
Stratum 6	55.5 (44.3, 67.4)	47.9 (38.7, 57.2)	57.4 (44.7, 70.7)
Stratum 7	60.7 (48.6, 73.3)	51.8 (41.9, 61.6)	63.7 (50.0, 78.1)
Stratum 8	66.5 (53.3, 80.2)	56.3 (45.7, 67.6)	71.1 (55.8, 87.5)
Stratum 9	73.8 (59.0, 89.5)	62.2 (49.3, 75.4)	81.1 (62.9, 100.8)
Stratum 10 (highest)	86.2 (67.1, 98.6)	72.7 (56.2, 83.0)	100.0 (73.5, 116.4)

Mean season-shifted 25(OH)D concentration in each stratum (nmol/L); in brackets, 10th and 90th percentiles of the distribution, or 20th and 80th percentiles for the lowest and highest strata.

## Data Availability

Data from UK Biobank is available to any *bona fide* scientific research on application. Applications to access data from EPIC-CVD should be addressed to the steering committee: https://www.phpc.cam.ac.uk/ceu/epic_cvd/. Data from the Vitamin D Studies Collaboration is available at the discretion of the principal investigators of the individual studies.

## Research in Context

### Evidence before this study

We searched for randomized trials investigating the effects of vitamin D supplementation on cardiovascular disease or all-cause mortality published in any language up until April 16, 2021 (with no specified earliest date), in PubMed, Scientific Citation Index Expanded, and Embase using the search terms listed in the appendix. However, no study had combined the following key features required to achieve reliable estimates of the effect of vitamin D supplementation on cardiovascular disease or mortality in vitamin D deficient individuals: availability of individual-participant data on large sample sizes (e.g., >1000 individuals with 25(OH)D <25 nmol/L) with moderate numbers of cardiovascular disease or all-cause mortality events (e.g., > 100 events). In summary, large randomized controlled trials have not been able to demonstrate beneficial effects of vitamin D supplementation on risk of cardiovascular disease or mortality.

### Added value of this study

We characterized dose—response relationships for 25-hydroxyvitamin D [25(OH)D], a metabolite used as a clinical indicator of vitamin D status, with cardiovascular disease and mortality in the largest such epidemiological analysis performed to date, using statistical methods tailored to the investigation of non-linear risk factor—disease relationships. Our observational analyses indicate threshold relationships between 25(OH)D concentrations and risk of both cardiovascular disease and all-cause mortality. However, genetic analyses did not support a causal effect of 25(OH)D concentrations on cardiovascular disease or mortality at any level of 25(OH)D concentrations.

### Implications of all the available evidence

Taken together, these findings suggest that long-term low-dose vitamin D supplementation is unlikely to reduce cardiovascular disease or mortality risk even in individuals with low 25(OH)D levels.
