# Supplementary material for: Estimating dose—response relationships for vitamin D with coronary heart disease, stroke, and all-cause mortality: observational and revised Mendelian randomization analyses
Source: Lancet Diabetes Endocrinol. Author manuscript; Available in PMC 2024 Feb 1. (PMC7615586; doi:10.1016/S2213-8587(23)00287-5)
Supplement: Supplementary material [file EMS193231-supplement-Supplementary_material.docx]

**Estimating dose—response relationships for vitamin D with coronary heart disease, stroke, and all-cause mortality: observational and Mendelian randomization analyses**

*Emerging Risk Factors Collaboration/EPIC-CVD/Vitamin D Studies Collaboration*

**CONTENTS**

Supplementary Methods 1-4
Literature Search Terms 5
Supplementary References 6
Supplementary Table 1: List of non-linear Mendelian randomization analyses of vitamin D performed using the residual stratification method 7
Supplementary Table 2: Methods for measurement of 25(OH)D concentration in studies of the Vitamin D Studies Collaboration 8
Supplementary Table 3: ICD-10 codes for cause-specific mortality outcomes 9
Supplementary Table 4: List of genetic variants for genetic risk score in UK Biobank and EPIC-CVD 10
Supplementary Table 5: Baseline characteristics of participants in the observational analyses 11
Supplementary Table 6: Details of studies contributing to the observational analyses 12-13
Supplementary Table 7: Distribution of 25(OH)D measurements and proportion of those with low 25(OH)D status in different seasons and months of blood draw in UK Biobank 14
Supplementary Table 8: Progressively adjusted observational associations of 25(OH)D concentrations with outcomes by clinical categories 15
Supplementary Table 9: Study-specific Mendelian randomization estimates for main outcomes in overall population and divided into clinical strata by residual concentration of 25(OH)D 16
Supplementary Table 10: Mendelian randomization estimates for stroke in UK Biobank 17
Supplementary Table 11: Mendelian randomization estimates for coronary heart disease in UK Biobank 18
Supplementary Table 12: Mendelian randomization estimates for main outcomes in UK Biobank 19
Supplementary Table 13: Study-specific Mendelian randomization estimates for cause-specific mortality 20
Supplementary Figure 1: Mean and spread of 25(OH)D measurements 21
Supplementary Figure 2: Associations of the focused (primary) genetic risk score for 25(OH)D concentrations with cardiovascular traits in UK Biobank 22
Supplementary Figure 3: Associations of the genome-wide score with cardiovascular traits in UK Biobank 23
Supplementary Figure 4: Observational associations of 25(OH)D concentrations with outcomes (age and sex adjusted only) 24
Supplementary Figure 5: Study-specific dose—response curves for associations of 25(OH)D concentrations 25
Supplementary Figure 6: Dose—response association of 25(OH)D concentrations with outcomes by deciles and categories 26
Supplementary Figure 7: Associations between the genetic risk score and 25(OH)D concentrations in strata of 25(OH)D concentrations 27
Supplementary Figure 8: Stratified Mendelian randomization estimates for mortality outcomes in UK Biobank from residual and doubly-ranked methods 28
List of Emerging Risk Factors Collaboration / Vitamin D Studies Collaboration Investigators 29

SUPPLEMENTARY METHODS

The stepwise selection method for selecting genetic variants to include in the genetic analyses had two components: a series of forward steps and then a backward step. In each forward step, genetic variants were ranked based on their associations with 25(OH)D concentrations conditional on variants selected at any previous step. At each step, the variant having the lowest p-value was selected. The process was repeated for each locus until no further variants were conditionally associated with 25(OH)D concentrations at a genome-wide level of significance (p < 5×10^-8^). Finally, a backward step was applied to omit any variant failing to meet the genome-wide level of significance for association with 25(OH)D concentrations in a joint model including all selected variants.

The adjusted analyses presented in Figure 1 include data on up to 431,489 individuals, comprising 67,992 from VitDSC, 14,941 from EPIC-CVD, and 348,556 from UK Biobank with complete data on all covariates. Associations for progressive levels of covariate adjustment are provided in Supplementary Table 8; each analysis is presented for the same complete data sample. Analysis for the full sample of 500,962 individuals with adjustment for age and sex only are presented in Supplementary Figure 4.

Outcome definitions for the Copenhagen studies were defined using ICD codes and manual validation for some outcomes as described previously [S1, S2, S3]. Only a limited number of genetic variants were available in the Copenhagen studies as these datasets have never been fully genotyped using a modern genetic chip.

To account for the case-cohort study design in EPIC-CVD, Cox models were adapted using Prentice weights and stratified by centre [S4]. To avoid overfitting models, studies contributing fewer than ten incident events to the analysis of a particular outcome were excluded from that analysis.

The primary dose—response analyses assessed the continuous shape of association of 25(OH)D and outcomes by meta-analysis of fractional polynomials adjusted for the conventional risk factors [S5]. First, the best-fitting fractional polynomial of degree 2 was estimated for each outcome using a Cox regression model fitted to the combined dataset stratified by study, centre, sex, and trial arm. Next, the coefficients for the best fitting fractional polynomial powers were estimated separately within each study, and then pooled across studies by random effects meta-analysis [S6]. The pooled coefficients were used to plot the continuous shape of association relative to the reference value of 50 nmol/L.

The rationale for the stratified genetic analyses (“non-linear Mendelian randomization”) has been explained at length previously [S7, S8]: briefly, exposure measurements are affected by the genetic variants used in the analyses as instrumental variables, so this represents a “post-randomization” covariate. Stratifying on the exposure directly would lead to collider bias, as the exposure is a collider (a common effect) of the genetic variants and confounders of the exposure—outcome association.

The doubly-ranked non-linear Mendelian randomization method first constructs subgroups of the population (“pre-strata”) that have similar levels of the instrument by ranking participants according to their level of the instrument, and then forms strata based on ranking participants according to their level of the exposure within each pre-stratum. The method assumes that the population ranking of individuals according to their exposure values would be similar at all values of the instrument (the “rank preserving assumption”). That is, the counterfactual values of the exposure for each individual would be at the same percentile of the exposure distribution (say, the 10^th^ percentile) whatever their value of the instrument. This assumption is strictly weaker than the “constant genetic effect” assumption made by the residual stratification method, as the constant genetic effect assumption is a specific case of the rank preserving assumption. However, the rank preserving assumption is more flexible, as it allows the effect of the genetic variant to be stronger or weaker at different levels of the exposure.

As stratum-specific estimates from the doubly-ranked method can be sensitive to specification of the analytic sample, we repeated analyses 100 times for each dataset omitting a small number of individuals in each iteration (12 individuals were removed at random in each iteration), and then combined estimates across iterations using Rubin’s rules.

In UK Biobank, after correcting for season of blood draw to convert all values to an autumn measurement, 16% of individuals had a 25(OH)D concentration below 35 nmol/L, and 25% below 40 nmol/L. A more detailed assessment of the distribution of 25(OH)D concentrations throughout the calendar year is provided in Supplementary Table 7.

Season correction in the genetic analyses was performed as follows. First, we calculated the average 25(OH)D measurement in each season, separately in each study (and centre for EPIC-CVD). Secondly, for measurements not taken in autumn, we subtracted the study-specific mean of measurements taken in that season, and added the study-specific mean of measurements taken in autumn. Spring is defined as March to May, summer is June to August, autumn is September to November, and winter is December to February. For illustration, let us assume that the mean value of 25(OH)D in a particular study is 50 nmol/L for participants measured in autumn, 70 nmol/L for participants measured in summer, and 40 nmol/L for participants measured in winter. To convert a winter measurement into an autumn measurement, we would add 10 nmol/L (50-40 = 10). To convert a summer measurement into an autumn measurement, we would subtract 20 nmol/L (50-70 = -20). So an individual with a summer measurement of 65 nmol/L would have a season-corrected value of 45 nmol/L.

The genome-wide score was derived as follows: from variants reported in Supplementary Table S2 of Manousaki *et al* [S9], we took one variant from each linkage disequilibrium block, selecting in each case the variant with the lowest p-value. Weights were taken as the beta-coefficients from the BOLT-LMM analysis in UK Biobank provided by the authors. In total, 71 variants were included in the genome-wide score. The genome-wide score explained 4.5% of the variance in 25(OH)D levels.

Some participants in the Copenhagen studies are included in the observational analyses as part of the Vitamin D Studies Collaboration. While there is overlap in participants between the observational and genetic analyses, we have been careful to avoid including participants twice within each study in either the observational analysis or the genetic analysis.

Derivation of the analytic sample for UK Biobank of individuals of European ancestries followed quality control steps described previously [S10]: after filtering genetic variants (call rate ≥ 99%, info score > 0.9, Hardy-Weinberg equilibrium p-value ≥ 10^-5^) and participants (removal of genetic sex mismatches), we excluded participants having non-European ancestries (self-report or inferred by genetics) or excess heterozygosity (>3 standard deviations from the mean), and included only one of each set of related participants (third-degree relatives or closer).

Estimates from the non-linear observational and genetic analyses have somewhat different interpretations. The observational analyses include incident events only. Estimates are hazard ratios relative to a common reference value – in main analyses, this is 50 nmol/L. The genetic analyses include both incident and prevalent events. Estimates are odds ratios and represent the association between genetically-predicted levels of the exposure (in our case, 25(OH)D concentrations) and the outcome. We scale estimates to correspond to a 10 nmol/L increase in genetically-predicted 25(OH)D concentration. Under the instrumental variable assumptions, overall estimates represent the population-averaged effect of a shift in the distribution of the exposure [S11]. In the non-linear Mendelian randomization analyses, estimates are odds ratios per 10 nmol/L increase in genetically-predicted 25(OH)D concentration calculated within a stratum of the population. Under the instrumental variable assumptions, estimates represent the stratum-averaged effect of a shift in the distribution of the exposure. While we use the term “non-linear Mendelian randomization” to connect to previous presentations of the methodology in the literature [S12], the term “stratified Mendelian randomization” may be more understandable, as the stratum-specific estimates are linear estimates, but estimated in a specific stratum of the population.

There are several differences between observational and genetic estimates: two key differences are that the observational estimates represent the association of current levels of 25(OH)D concentrations with disease risk, whereas genetic estimates represent the association of genetically-predicted levels of 25(OH)D concentrations with disease risk, hence reflecting the impact of long-term differences in 25(OH)D levels. Another difference is that the genetic analyses are conducted separately within each stratum, and so there is no common reference category; estimates represent the impact of 10 nmol/L higher genetically-predicted 25(OH)D concentrations in each stratum.

For the genetic analyses, all 25(OH)D measurements were standardized by quality control as certified by the Vitamin D Standardization-Certification Program of the Centers for Disease Control and Prevention (VDSP) for UK Biobank, or the Vitamin D External Quality Assessment Scheme (DEQAS) for EPIC-CVD and the Copenhagen studies. Several cohorts in VitDSC also measured 25(OH)D in accredited laboratories.

Our literature search terms were as follows:

**PubMed, search through 16 April 2021**

("Vitamin D"[Mesh] OR "Vitamin D" OR "25-hydroxyvitamin D" OR "25(OH)D" OR "Calciferol" OR "Vitamin D2" OR "Vitamin D3" OR "Cholecalciferol" OR "Ergocalciferol" OR "Alphacalcidol" OR "Alfacalcidol" OR "Calcitriol" OR "Paricalcitol" OR "Doxerocalciferol")

AND ("Cardiovascular Diseases"[Mesh] OR "Cardiovascular Disease" OR "All-cause Mortality" OR "Mortality" OR "Survival")

AND ("Randomized Controlled Trial" [Publication Type] OR "Randomized Controlled Trials as Topic"[Mesh] OR "Randomized Controlled Trial" OR "Controlled Clinical Trial" [Publication Type] OR "Controlled Clinical Trials as Topic"[Mesh] OR "Controlled Clinical Trial" OR "Random Allocation" OR "Trial")

**Scientiﬁc Citation Index Expanded, search through 16 April 2021**

TS= ("Vitamin D" OR "25-hydroxyvitamin D" OR "25(OH)D" OR "Calciferol" OR "Vitamin D2" OR "Vitamin D3" OR "Cholecalciferol" OR "Ergocalciferol" OR "Alphacalcidol" OR "Alfacalcidol" OR "Calcitriol" OR "Paricalcitol" OR "Doxerocalciferol") AND TS= ("Cardiovascular Disease" OR "All-cause Mortality" OR "Mortality" OR "Survival")

AND TS= ("Randomized Controlled Trial" OR "Controlled Clinical Trial" OR "Random Allocation" OR "Trial")

**EMBASE, search through 15 April 2021**

("Vitamin D" OR "25-hydroxyvitamin D" OR "25(OH)D" OR "Calciferol" OR "Vitamin D2" OR "Vitamin D3" OR "Cholecalciferol" OR "Ergocalciferol" OR "Alphacalcidol" OR "Alfacalcidol" OR "Calcitriol" OR "Paricalcitol" OR "Doxerocalciferol").af. AND ("Cardiovascular Disease" OR "All-cause Mortality" OR "Mortality" OR "Survival").af. AND ("Randomized Controlled Trial" OR "Controlled Clinical Trial" OR "Random Allocation" OR "Trial").af. (Limited to Embase Status)

In total, we identified 90 relevant articles, including 79 studies of all-cause mortality and 41 studies of cardiovascular outcomes.

Supplementary references:

S1. Afzal S, Brøndum-Jacobsen P, Bojesen SE, Nordestgaard BG. Genetically low vitamin D concentrations and increased mortality: mendelian randomisation analysis in three large cohorts. British Medical Journal 2014. **349**:g6330.

S2. Riis J, Nordestgaard BG, Jensen BG, Afzal S. Secular trends in risk of stroke according to body mass index and blood pressure, 1976–2017. Neurology 2019. **93**(14):e1397-e1407.

S3. Brøndum-Jacobsen P, Benn M, Jensen GB, Nordestgaard BG. 25-hydroxyvitamin d levels and risk of ischemic heart disease, myocardial infarction, and early death: population-based study and meta-analyses of 18 and 17 studies. Arteriosclerosis, Thrombosis, and Vascular Biology 2012. **32**(11):2794-802.

S4. Prentice RL. A case-cohort design for epidemiologic cohort studies and disease prevention trials. Biometrika, 1986. **73**(1):1-11.

S5. White IR, Kaptoge S, Royston P, Sauerbrei W, ERFC*.* Meta‐analysis of non‐linear exposure‐outcome relationships using individual participant data: a comparison of two methods. Statistics in Medicine, 2019. **38**(3):326-338.

S6. Thompson SG, Kaptoge S, White I, Wood A, Perry P, Danesh J, ERFC*.* Statistical methods for the time-to-event analysis of individual participant data from multiple epidemiological studies. International Journal of Epidemiology, 2010. **39**(5):1345-1359.

S7. Tian H, Burgess S. Relaxing parametric assumptions for non-linear Mendelian randomization using a doubly-ranked stratification method. PLOS Genetics 2023 (in press).

S8. Burgess S. Violation of the constant genetic effect assumption can result in biased estimates for non-linear Mendelian randomization. Human Heredity 2023 (in press).

S9. Manousaki D, Mitchell R, Dudding T, et al. Genome-wide association study for vitamin D levels reveals 69 independent loci. The American Journal of Human Genetics 2020; **106**(3):327-337.

S10. Astle WJ, Elding H, Jiang T, et al. The allelic landscape of human blood cell trait variation and links to common complex disease. Cell 2016; **167**(5):1415-1429.

S11. Burgess S, Davies NM, Thompson SG. Instrumental variable analysis with a nonlinear exposure–outcome relationship. Epidemiology 2014; **25**(6):877-885.

S12. Sun Y-Q, Burgess S, Staley JR, et al. Body mass index and all cause mortality in HUNT and UK Biobank studies: linear and non-linear mendelian randomisation analyses. British Medical Journal 2019; **364**:l1042.S12.

SUPPLEMENTARY TABLES AND FIGURES

**Supplementary Table 1:** List of non-linear Mendelian randomization analyses of vitamin D performed using the residual stratification method

| **Lead author** | **Journal and year** | **Link** |
| --- | --- | --- |
| Sofianopoulou | Lancet Diabetes Endocrinol 2021 | https://pubmed.ncbi.nlm.nih.gov/34717822/ |
| Zhou | Euro Heart J 2022 | https://pubmed.ncbi.nlm.nih.gov/34891159/ |
| Sutherland | Ann Intern Med 2022 | https://pubmed.ncbi.nlm.nih.gov/36279545/ |
| Navale | Am J Clin Nutr 2022 | https://pubmed.ncbi.nlm.nih.gov/35451454/ |
| Zhou | Int J Epidemiol 2023 | https://pubmed.ncbi.nlm.nih.gov/35579027/ |
| Sutherland* | Nutrients 2023 | https://pubmed.ncbi.nlm.nih.gov/37375607/ |
| Sha | JAMA Network Open 2023 | https://pubmed.ncbi.nlm.nih.gov/37647062/ |

* Sutherland used both the residual and doubly-ranked stratification methods, obtaining markedly different results from the two methods.

**Supplementary Table 2:** Methods for measurement of 25(OH)D concentration in studies of the Vitamin D Studies Collaboration

| Method | Number of studies |
| --- | --- |
| Radioimmunoassay (RIA) | 11 |
| Automated immunoassay | 4 |
| Competitive protein binding (CPB) | 1 |
| Immunometric assay (IMA) | 1 |
| High-performance liquid chromatography mass spectrometry (HPLC-MS) | 13 |
| Electro-chemiluminescence immunoassay (ECLIA) | 1 |

A list of the methods used in each study is provided in Supplementary Table 5.

**Supplementary Table 3:** ICD-10 codes for cause-specific mortality outcomes

| Cause-specific mortality | ICD-10 codes |
| --- | --- |
| Cardiovascular | G45, I01, I03-I82, I87, I95-I99, F01, Q20-Q28, R96 |
| Cancer | C00-C97, D00-D48 |
| Non-cardiovascular non-cancer | All others |

**Supplementary Table 4:** List of genetic variants for genetic risk score in UK Biobank and EPIC-CVD

| Chromosome: Position (hg19) | rsID | Effect allele | Other allele | Conditional association with 25(OH)D (nmol/L) |
| --- | --- | --- | --- | --- |
| 4:72617775 | rs1352846 | G | A | 0.172 |
| 4:72618334 | rs7041 | C | A | -0.045 |
| 4:72634343 | rs4694431 | T | C | -0.034 |
| 4:72770563 | rs139148694 | GTGCTTTTATCAA | G | 0.028 |
| 11:14339328 | rs16913816 | A | G | -0.031 |
| 11:14900931 | rs117913124 | A | G | 0.503 |
| 11:14912573 | rs117576073 | T | G | 0.246 |
| 11:14913575 | rs12794714 | A | G | 0.139 |
| 11:14913645 | rs202122669 | A | G | -0.615 |
| 11:14913900 | rs187639972 | C | G | -0.360 |
| 11:14941652 | rs117115472 | G | C | 0.148 |
| 11:71157867 | rs139168803 | A | G | -0.188 |
| 11:71158672 | rs12573951 | G | A | -0.045 |
| 11:71161063 | rs7928249 | G | A | -0.131 |
| 11:71180762 | rs549000212 | A | C | -0.364 |
| 11:71290740 | rs4081429 | C | A | 0.017 |
| 20:52714706 | rs6123359 | G | A | -0.026 |
| 20:52731402 | rs6127099 | T | A | 0.013 |
| 20:52735238 | rs35870583 | GT | G | 0.027 |
| 20:52737123 | rs2585442 | G | C | -0.025 |
| 20:52788925 | rs2762942 | A | G | -0.053 |

In the Copenhagen studies, rs12794714 (11:14913575) and rs117913124 (11:14900931), and rs7944926 (11:71165625) were used for genetic analyses. The rs7944926 variant is in high linkage disequilibrium with rs7928249 (r^2^ = 0.986 in European ancestry 1000 Genomes participants).

**Supplementary Table 5:** Baseline characteristics of participants in the observational analyses

| Characteristic* | Cohorts | N | Mean (SD) or % |
| --- | --- | --- | --- |
| 25(OH)D (nmol/L) | 40 | 500,962 | 52.0 (21.7) |
| <25 nmol/L, Deficient | 40 | 65,313 | 13% |
| 25-49 nmol/L, Insufficient | 40 | 208,223 | 42% |
| 50-74 nmol/L, Sufficient | 40 | 165,162 | 33% |
| ≥75 nmol/L, Adequate | 40 | 62,264 | 12% |
| **Age and physical measures** |  |  |  |
| Age at survey (yrs) | 40 | 500,962 | 60.7 (8.7) |
| Height (cm) | 40 | 499,369 | 167 (9) |
| Weight (kg) | 40 | 498,356 | 74.0 (15.4) |
| Body mass index [BMI] (kg/m^2) | 40 | 498,019 | 26.6 (4.7) |
| Waist circumference (cm) | 29 | 464,017 | 89.0 (13.2) |
| Hip circumference (cm) | 29 | 463,946 | 102 (9) |
| Waist:Hip circumference ratio | 29 | 463,881 | 0.87 (0.09) |
| Systolic blood pressure [SBP] (mmHg) | 38 | 488,928 | 138 (19) |
| Diastolic blood pressure [DBP] (mmHg) | 37 | 486,117 | 80.2 (10.3) |
| **Lipids** |  |  |  |
| Total cholesterol (mmol/l) | 37 | 489,120 | 5.92 (1.02) |
| Friedewald LDL cholesterol (mmol/l) | 32 | 431,296 | 3.80 (0.89) |
| Measured LDL cholesterol (mmol/l) | 3 | 388,309 | 3.37 (0.76) |
| Non-HDL cholesterol (mmol/l) | 35 | 452,863 | 4.49 (1.00) |
| HDL-cholesterol (mmol/l) | 35 | 453,646 | 1.40 (0.38) |
| Log Triglycerides (mmol/l) | 34 | 476,028 | 0.34 (0.52) |
| Apolipoprotein A1 (g/l) | 9 | 374,898 | 1.51 (0.27) |
| Apolipoprotein B (g/l) | 9 | 407,933 | 1.05 (0.22) |
| Log Lipoprotein a [Lp(a)] (mg/dl) | 9 | 329,672 | 3.18 (1.11) |
| **Glycaemia markers** |  |  |  |
| Log Glucose (mmol/l) | 29 | 426,373 | 1.65 (0.19) |
| Log Fasting glucose (mmol/l) | 16 | 39,327 | 1.64 (0.19) |
| Glycated haemoglobin [HbA1c] (%) | 9 | 389,733 | 5.52 (0.86) |
| **Inflammation markers** |  |  |  |
| Fibrinogen (µmol/l) | 12 | 24,246 | 9.15 (2.27) |
| Log C-reactive protein [CRP] (mg/l) | 29 | 447,320 | 0.43 (1.06) |
| Log White cell count (x10^9/l) | 13 | 400,868 | 1.85 (0.26) |
| Albumin (g/l) | 25 | 411,035 | 43.7 (2.8) |
| **Kidney function** |  |  |  |
| Log Creatinine (µmol/l) | 30 | 446,152 | 4.40 (0.20) |
| Log eGFR by MDRD (ml/min/1.73m^2) | 30 | 446,152 | 4.31 (0.20) |
| **Bone-related markers** |  |  |  |
| Calcium (mmol/l) | 21 | 401,249 | 2.38 (0.10) |
| Log Parathyroid hormone (ng/L) | 14 | 21,379 | 1.30 (0.44) |
| Log Phosphate (mmol/L) | 13 | 365,621 | 0.13 (0.14) |
| Log Calcitriol [1,25(OH)_2_D] (pmol/L) | 7 | 3,160 | 4.38 (0.42) |
| Log Alkaline Phosphatase (IU/L) | 10 | 419,940 | 4.16 (0.28) |
| **Categorical variables** |  |  |  |
| Sex | 40 | 500,962 |  |
| Male | 32 | 225,383 | 45% |
| Female | 36 | 275,579 | 55% |
| Ethnic group (4 groups) | 36 | 489,927 |  |
| White | 36 | 463,935 | 95% |
| Asian | 7 | 8,504 | 2% |
| Black | 8 | 10,749 | 2% |
| Other | 8 | 6,739 | 1% |
| Smoking status | 40 | 499,495 |  |
| Other | 40 | 431,683 | 86% |
| Current | 40 | 67,812 | 14% |
| Alcohol status | 34 | 481,755 |  |
| Other | 34 | 54,161 | 11% |
| Current | 31 | 427,594 | 89% |
| History of diabetes | 40 | 488,586 |  |
| No | 39 | 465,183 | 95% |
| Yes | 40 | 23,403 | 5% |
| Season of 25(OH)D blood draw | 40 | 500,962 |  |
| Winter | 36 | 99,694 | 20% |
| Spring | 35 | 138,518 | 28% |
| Summer | 39 | 142,646 | 28% |
| Autumn | 38 | 120,104 | 24% |
| Highest level of education reached | 28 | 456,164 |  |
| Primary | 25 | 25,375 | 6% |
| Secondary | 28 | 176,416 | 39% |
| Vocational/University | 24 | 254,373 | 56% |

* Common abbreviations are shown in square brackets: LDL low density lipoprotein cholesterol; HDL high-density lipoprotein cholesterol; eGFR estimated glomerular filtration rate using the Modification of Diet in Renal Disease (MDRD) equation. For the purposes of this table, EPIC-CVD countries are enumerated as separate cohorts as covariate information differed by centre.

**Supplementary Table 6:** Details of studies contributing to the observational analyses

**Supplementary Table 6 (continued):** List of acronyms of studies contributing to the observational analyses.

| Cohort abbreviation | Cohort name | 25(OH)D assay method |
| --- | --- | --- |
| 4D | The German Diabetes & Dialysis Study | Automated immunoassay |
| AUCKLAND | Auckland Calcium Study | RIA |
| BRUN | Bruneck Study | Automated immunoassay |
| BWHHS | British Women's Heart and Health Study | HPLC-MS |
| CAIFOS | Calcium Intake Fracture Outcome Study | HPLC-MS |
| CAPS | Caerphilly Prospective Study | HPLC-MS |
| CCHS | Copenhagen City Heart Study | RIA |
| DOPS | Danish Osteoporosis Prevention Study | RIA |
| EPICBMD | European Prospective Investigation of Cancer - Norfolk Study (Bone Mineral Density sub-study) | IMA |
| EPICNOR | European Prospective Investigation of Cancer - Norfolk Study | HPLC-MS |
| ESTHER | Epidemiologische Studie zu Chancen der Verhütung und optimierten Therapie chronischer Erkrankungen in der älteren Bevölkerung | RIA |
| HCS | Hertfordshire Cohort Study | RIA |
| HDZNRW | The Heart and Diabetes Center NRW Study | RIA |
| INTER99 | Inter99 Study | HPLC-MS |
| LASA | Longitudinal Aging Study Amsterdam | CPB |
| LURIC | Ludwigshafen Risk and Cardiovascular Health | RIA |
| MESA | Multi-Ethnic Study of Atherosclerosis | HPLC-MS |
| MIDSPAN | MIDSPAN Family Study | HPLC-MS |
| MINIFIN | Mini-Finland Health Survey | RIA |
| MONICA10 | Monitoring of Trends and Determinants in Cardiovascular Disease | ECLIA |
| MROS | Osteoporotic Fractures in Men | HPLC-MS |
| NHANESIII | Third National Health and Nutrition Examination Survery | RIA |
| PROSPER | Prospective Study of Pravastatin in the Elderly at Risk | HPLC-MS |
| SHIP-1 | Study of Health in Pomerania-1 | Automated immunoassay |
| SOF1 | Study of Osteoporotic Fractures (visit 1) | HPLC-MS |
| SOF4 | Study of Osteoporotic Fractures (visit 4) | HPLC-MS |
| STENO | The Steno Diabetes Study | HPLC-MS |
| TURKUFIN | Turku-Finland Elderly Study | RIA |
| TWINSUK | Twins UK Study | RIA |
| ULSAM | The Uppsala Longitudinal Study of Adult Men | HPLC-MS |
| WHITEI | Whitehall I | Automated immunoassay |
| EPICCVD_Denmark | EPIC-CVD Denmark (Aarhus, Copenhagen) | HPLC-MS |
| EPICCVD_France | EPIC-CVD France (France) | HPLC-MS |
| EPICCVD_Germany | EPIC-CVD Germany (Heidelberg, Potsdam) | HPLC-MS |
| EPICCVD_Italy | EPIC-CVD Italy (Florence, Varese, Ragusa, Turin, Naples) | HPLC-MS |
| EPICCVD_Netherlands | EPIC-CVD Netherlands (Bilthoven, Utrecht) | HPLC-MS |
| EPICCVD_Spain | EPIC-CVD Spain (Asturias, Granada, Murcia, Navarra, San Sebastian) | HPLC-MS |
| EPICCVD_Sweden | EPIC-CVD Sweden (Umea) | HPLC-MS |
| EPICCVD_UK | EPIC-CVD UK (Cambridge, Oxford) | HPLC-MS |
| UKBIOBANK | UK Biobank | CLIA |

The EPIC-CVD study was specifically designed as a case-cohort study of CVD outcomes therefore does not contribute to the analysis of non-CVD outcomes nor all-cause mortality.

**Supplementary Table 7:** Distribution of 25(OH)D measurements and proportion of those with low 25(OH)D status in different seasons and months of blood draw in UK Biobank.

| Season | Winter | Spring | Summer | Autumn |
| --- | --- | --- | --- | --- |
| Mean 25(OH)D (nmol/L) | 41.0 | 42.9 | 58.2 | 54.5 |
| Participants below 25 nmol/L (%) | 22.2 | 19.1 | 2.9 | 6.0 |
| Participants below 40 nmol/L (%) | 54.2 | 49.9 | 17.4 | 25.6 |

|  | Winter | | | Spring | | | Summer | | | Autumn | | |
| --- | --- | --- | --- | --- | --- | --- | --- | --- | --- | --- | --- | --- |
| Month | Dec | Jan | Feb | Mar | Apr | May | Jun | Jul | Aug | Sep | Oct | Nov |
| Mean 25(OH)D | 44.4 | 40.1 | 39.4 | 39.5 | 42.0 | 47.0 | 55.1 | 59.7 | 60.7 | 60.0 | 55.4 | 48.5 |
| % below 25 nmol/L | 15.7 | 23.1 | 25.8 | 26.1 | 20.5 | 11.2 | 3.9 | 2.5 | 2.1 | 2.6 | 4.8 | 10.3 |
| % below 40 nmol/L | 46.5 | 56.0 | 58.0 | 58.1 | 52.7 | 39.8 | 21.8 | 15.4 | 13.8 | 15.5 | 23.3 | 37.1 |

**Supplementary Table 8:** Progressively adjusted observational associations of 25(OH)D concentrations with outcomes by clinical categories. The adequate stratum is the reference group. Confidence intervals are calculated using the floating variance method. Each analysis is performed for the same data sample with complete data on all covariates.

| **Outcome / Progressive adjustment variables** | **Hazard ratio (95% confidence interval)** | | | |
| --- | --- | --- | --- | --- |
| **(N = Cohorts / Participants / Outcomes)** | Deficient  (< 25 nmol/L) | Insufficient  (25-49 nmol/L) | Sufficient  (50-74 nmol/L) | Adequate [Ref]  (≥ 75 nmol/L) |
| **Coronary heart disease**  **(N = 31 / 417,937 / 12,818)** |  |  |  |  |
| Adjusted for sex and age | 1.38 (1.22, 1.55) | 1.11 (1.05, 1.18) | 1.02 (0.96, 1.08) | 1.00 (0.91, 1.10) |
| Plus month of recruitment | 1.48 (1.32, 1.66) | 1.16 (1.09, 1.23) | 1.04 (1.01, 1.08) | 1.00 (0.91, 1.10) |
| Plus smoking status | 1.34 (1.22, 1.47) | 1.12 (1.09, 1.14) | 1.03 (0.97, 1.09) | 1.00 (0.88, 1.13) |
| Plus total cholesterol | 1.37 (1.24, 1.52) | 1.12 (1.08, 1.17) | 1.03 (0.97, 1.09) | 1.00 (0.89, 1.12) |
| Plus HDL cholesterol | 1.25 (1.12, 1.39) | 1.04 (0.98, 1.10) | 0.99 (0.94, 1.04) | 1.00 (0.89, 1.12) |
| Plus systolic blood pressure | 1.21 (1.07, 1.36) | 1.02 (0.96, 1.09) | 0.98 (0.94, 1.01) | 1.00 (0.90, 1.11) |
| Plus history of diabetes | 1.17 (1.05, 1.30) | 1.00 (0.95, 1.06) | 0.97 (0.93, 1.02) | 1.00 (0.89, 1.12) |
| Plus body mass index | 1.16 (1.04, 1.29) | 1.00 (0.94, 1.06) | 0.97 (0.93, 1.01) | 1.00 (0.89, 1.12) |
| **Stroke**  **(N = 30 / 427,698 / 9947)** |  |  |  |  |
| Adjusted for sex and age | 1.48 (1.37, 1.61) | 1.17 (1.09, 1.27) | 1.01 (0.95, 1.07) | 1.00 (0.94, 1.07) |
| Plus month of recruitment | 1.58 (1.44, 1.73) | 1.20 (1.10, 1.31) | 1.02 (0.95, 1.09) | 1.00 (0.94, 1.07) |
| Plus smoking status | 1.50 (1.37, 1.63) | 1.17 (1.07, 1.27) | 1.01 (0.94, 1.07) | 1.00 (0.94, 1.07) |
| Plus total cholesterol | 1.50 (1.38, 1.64) | 1.17 (1.08, 1.27) | 1.01 (0.95, 1.08) | 1.00 (0.94, 1.07) |
| Plus HDL cholesterol | 1.45 (1.33, 1.58) | 1.14 (1.05, 1.24) | 1.00 (0.93, 1.06) | 1.00 (0.93, 1.07) |
| Plus systolic blood pressure | 1.40 (1.28, 1.53) | 1.12 (1.03, 1.22) | 0.99 (0.92, 1.06) | 1.00 (0.93, 1.08) |
| Plus history of diabetes | 1.36 (1.25, 1.49) | 1.10 (1.01, 1.19) | 0.97 (0.91, 1.04) | 1.00 (0.93, 1.08) |
| Plus body mass index | 1.36 (1.24, 1.49) | 1.10 (1.01, 1.19) | 0.97 (0.90, 1.04) | 1.00 (0.93, 1.08) |
| **All-cause mortality**  **(N = 26 / 416,548 / 36,949)** |  |  |  |  |
| Adjusted for sex and age | 1.76 (1.60, 1.93) | 1.24 (1.19, 1.29) | 1.03 (1.00, 1.07) | 1.00 (0.98, 1.02) |
| Plus month of recruitment | 1.89 (1.71, 2.09) | 1.28 (1.24, 1.34) | 1.04 (1.01, 1.08) | 1.00 (0.98, 1.02) |
| Plus smoking status | 1.73 (1.57, 1.91) | 1.24 (1.20, 1.29) | 1.04 (1.00, 1.08) | 1.00 (0.98, 1.02) |
| Plus total cholesterol | 1.75 (1.58, 1.93) | 1.26 (1.21, 1.31) | 1.05 (1.01, 1.09) | 1.00 (0.98, 1.02) |
| Plus HDL cholesterol | 1.74 (1.57, 1.92) | 1.25 (1.20, 1.30) | 1.05 (1.01, 1.08) | 1.00 (0.98, 1.02) |
| Plus systolic blood pressure | 1.72 (1.55, 1.91) | 1.24 (1.19, 1.30) | 1.04 (1.01, 1.08) | 1.00 (0.98, 1.02) |
| Plus history of diabetes | 1.68 (1.51, 1.86) | 1.22 (1.16, 1.28) | 1.03 (1.00, 1.07) | 1.00 (0.98, 1.02) |
| Plus body mass index | 1.66 (1.50, 1.83) | 1.21 (1.15, 1.26) | 1.03 (0.99, 1.06) | 1.00 (0.98, 1.02) |
| **Cardiovascular mortality**  **(N = 33 / 431,489 / 9953)** |  |  |  |  |
| Adjusted for sex and age | 1.92 (1.72, 2.15) | 1.36 (1.33, 1.40) | 1.05 (1.00, 1.11) | 1.00 (0.93, 1.07) |
| Plus month of recruitment | 2.07 (1.83, 2.35) | 1.42 (1.38, 1.46) | 1.07 (1.03, 1.12) | 1.00 (0.94, 1.06) |
| Plus smoking status | 1.92 (1.70, 2.17) | 1.37 (1.34, 1.41) | 1.07 (1.02, 1.11) | 1.00 (0.94, 1.06) |
| Plus total cholesterol | 1.94 (1.72, 2.19) | 1.39 (1.35, 1.43) | 1.07 (1.03, 1.12) | 1.00 (0.94, 1.06) |
| Plus HDL cholesterol | 1.87 (1.65, 2.12) | 1.35 (1.31, 1.39) | 1.06 (1.01, 1.11) | 1.00 (0.94, 1.06) |
| Plus systolic blood pressure | 1.80 (1.59, 2.04) | 1.31 (1.28, 1.35) | 1.05 (1.00, 1.09) | 1.00 (0.94, 1.06) |
| Plus history of diabetes | 1.74 (1.54, 1.98) | 1.27 (1.23, 1.31) | 1.03 (0.99, 1.07) | 1.00 (0.94, 1.06) |
| Plus body mass index | 1.67 (1.48, 1.88) | 1.22 (1.19, 1.26) | 1.01 (0.97, 1.05) | 1.00 (0.94, 1.07) |
| **Cancer mortality**  **(N = 22 / 407,567 / 14,581)** |  |  |  |  |
| Adjusted for sex and age | 1.38 (1.26, 1.52) | 1.21 (1.15, 1.26) | 1.01 (1.00, 1.03) | 1.00 (0.95, 1.05) |
| Plus month of recruitment | 1.50 (1.39, 1.63) | 1.24 (1.19, 1.30) | 1.02 (1.00, 1.04) | 1.00 (0.95, 1.05) |
| Plus smoking status | 1.40 (1.31, 1.49) | 1.20 (1.14, 1.26) | 1.01 (0.99, 1.03) | 1.00 (0.95, 1.05) |
| Plus total cholesterol | 1.42 (1.33, 1.51) | 1.21 (1.15, 1.27) | 1.02 (0.99, 1.04) | 1.00 (0.95, 1.05) |
| Plus HDL cholesterol | 1.39 (1.30, 1.48) | 1.19 (1.13, 1.24) | 1.01 (0.99, 1.04) | 1.00 (0.95, 1.05) |
| Plus systolic blood pressure | 1.37 (1.27, 1.47) | 1.18 (1.13, 1.23) | 1.01 (0.98, 1.03) | 1.00 (0.95, 1.05) |
| Plus history of diabetes | 1.37 (1.29, 1.46) | 1.17 (1.12, 1.23) | 1.00 (0.98, 1.03) | 1.00 (0.95, 1.05) |
| Plus body mass index | 1.38 (1.31, 1.45) | 1.15 (1.11, 1.20) | 1.00 (0.98, 1.03) | 1.00 (0.95, 1.05) |
| **Non-cardiovascular/cancer mortality**  **(N = 23 / 408,536 / 9662)** |  |  |  |  |
| Adjusted for sex and age | 2.10 (1.80, 2.45) | 1.29 (1.21, 1.38) | 0.99 (0.91, 1.08) | 1.00 (0.96, 1.04) |
| Plus month of recruitment | 2.31 (1.93, 2.77) | 1.36 (1.25, 1.48) | 0.99 (0.92, 1.08) | 1.00 (0.97, 1.03) |
| Plus smoking status | 2.09 (1.76, 2.48) | 1.31 (1.21, 1.43) | 0.99 (0.91, 1.07) | 1.00 (0.97, 1.03) |
| Plus total cholesterol | 2.14 (1.78, 2.57) | 1.34 (1.22, 1.47) | 1.00 (0.92, 1.09) | 1.00 (0.97, 1.03) |
| Plus HDL cholesterol | 2.20 (1.81, 2.67) | 1.38 (1.25, 1.52) | 1.03 (0.95, 1.10) | 1.00 (0.97, 1.03) |
| Plus systolic blood pressure | 2.18 (1.79, 2.67) | 1.37 (1.24, 1.52) | 1.03 (0.95, 1.10) | 1.00 (0.97, 1.03) |
| Plus history of diabetes | 2.12 (1.75, 2.57) | 1.34 (1.21, 1.50) | 1.01 (0.93, 1.09) | 1.00 (0.98, 1.02) |
| Plus body mass index | 2.11 (1.75, 2.54) | 1.34 (1.20, 1.49) | 1.01 (0.93, 1.09) | 1.00 (0.98, 1.03) |

**Supplementary Table 9:** Study-specific Mendelian randomization estimates for main outcomes in overall population and divided into clinical strata by residual concentration of 25(OH)D.

| **Study and stratum** | **Mean 25(OH)D (nmol/L)** | **Coronary heart disease** | **Stroke** | **All cause mortality** |
| --- | --- | --- | --- | --- |
| *UK Biobank* |  |  |  |  |
| Overall |  | 0.98 (0.95, 1.01) | 1.01 (0.96, 1.05) | 1.00 (0.96, 1.03) |
| Stratum 1 | 28.6 | 1.32 (0.98, 1.77) | 1.10 (0.75, 1.63) | 1.06 (0.81, 1.39) |
| Stratum 2 | 35.7 | 0.94 (0.74, 1.19) | 1.24 (0.90, 1.73) | 1.06 (0.84, 1.34) |
| Stratum 3 | 41.1 | 0.98 (0.80, 1.20) | 1.06 (0.81, 1.39) | 1.09 (0.89, 1.33) |
| Stratum 4 | 46.0 | 1.02 (0.86, 1.22) | 1.04 (0.82, 1.33) | 1.12 (0.94, 1.34) |
| Stratum 5 | 50.7 | 0.93 (0.81, 1.07) | 0.94 (0.77, 1.14) | 0.95 (0.82, 1.10) |
| Stratum 6 | 55.5 | 0.96 (0.85, 1.09) | 0.92 (0.77, 1.09) | 0.98 (0.86, 1.11) |
| Stratum 7 | 60.7 | 0.97 (0.86, 1.08) | 0.91 (0.79, 1.06) | 0.93 (0.83, 1.04) |
| Stratum 8 | 66.5 | 0.95 (0.87, 1.04) | 0.95 (0.84, 1.08) | 0.97 (0.88, 1.07) |
| Stratum 9 | 73.8 | 0.97 (0.89, 1.05) | 1.02 (0.91, 1.14) | 0.97 (0.89, 1.05) |
| Stratum 10 | 86.2 | 1.00 (0.94, 1.07) | 1.07 (0.97, 1.17) | 1.01 (0.94, 1.08) |
| *EPIC-CVD* |  |  |  |  |
| Overall |  | 1.00 (0.91, 1.10) | 1.10 (0.96, 1.26) |  |
| Stratum 1 | 25.3 | 0.97 (0.45, 2.10) | 1.17 (0.57, 2.39) |  |
| Stratum 2 | 31.9 | 0.86 (0.48, 1.54) | 1.18 (0.66, 2.10) |  |
| Stratum 3 | 36.4 | 1.38 (0.86, 2.22) | 1.08 (0.65, 1.77) |  |
| Stratum 4 | 40.3 | 0.91 (0.60, 1.39) | 0.96 (0.61, 1.49) |  |
| Stratum 5 | 44.1 | 1.04 (0.70, 1.55) | 0.95 (0.66, 1.37) |  |
| Stratum 6 | 47.9 | 0.97 (0.70, 1.35) | 1.03 (0.72, 1.48) |  |
| Stratum 7 | 51.8 | 0.93 (0.69, 1.24) | 0.96 (0.70, 1.32) |  |
| Stratum 8 | 56.3 | 0.81 (0.62, 1.08) | 0.83 (0.64, 1.08) |  |
| Stratum 9 | 62.2 | 1.02 (0.79, 1.31) | 0.91 (0.73, 1.15) |  |
| Stratum 10 | 72.7 | 0.89 (0.74, 1.07) | 1.02 (0.85, 1.22) |  |
| *Copenhagen studies* |  |  |  |  |
| Overall |  | 0.95 (0.86, 1.05) | 0.99 (0.90, 1.09) | 0.89 (0.80, 0.99) |
| Stratum 1 | 25.3 | 0.94 (0.69, 1.28) | 0.93 (0.59, 1.45) | 0.95 (0.66, 1.37) |
| Stratum 2 | 33.6 | 0.91 (0.64, 1.30) | 0.91 (0.56, 1.48) | 0.92 (0.60, 1.41) |
| Stratum 3 | 40.1 | 0.98 (0.68, 1.41) | 0.97 (0.56, 1.67) | 0.91 (0.60, 1.38) |
| Stratum 4 | 46.0 | 0.99 (0.67, 1.45) | 1.00 (0.59, 1.70) | 0.93 (0.61, 1.42) |
| Stratum 5 | 51.6 | 0.93 (0.64, 1.36) | 1.15 (0.63, 2.09) | 0.93 (0.60, 1.44) |
| Stratum 6 | 57.4 | 0.92 (0.63, 1.35) | 1.27 (0.72, 2.24) | 0.90 (0.57, 1.43) |
| Stratum 7 | 63.7 | 1.04 (0.70, 1.55) | 1.35 (0.74, 2.45) | 0.88 (0.57, 1.38) |
| Stratum 8 | 71.1 | 1.10 (0.73, 1.67) | 1.29 (0.70, 2.37) | 0.81 (0.51, 1.28) |
| Stratum 9 | 81.1 | 1.14 (0.76, 1.73) | 1.09 (0.63, 1.89) | 0.81 (0.52, 1.27) |
| Stratum 10 | 100.0 | 1.11 (0.76, 1.61) | 1.31 (0.75, 2.28) | 0.83 (0.56, 1.23) |

Estimates (95% confidence intervals) represent odds ratio per 10 nmol/L higher genetically-predicted concentration of 25(OH)D.

**Supplementary Table 10:** Mendelian randomization estimates for stroke in UK Biobank divided into overall stroke (10,489 events), incident-only stroke (excluding those with prevalent stroke at baseline, 5044 events), ischaemic stroke (including unknown, 4164 events), and haemorrhagic stroke (intracerebral plus subarachnoid haemorrhage, 1194 events): odds ratios (95% confidence intervals) per 10 nmol/L higher genetically-predicted concentration of 25(OH)D.

| Stroke | Overall | Incident only | Ischaemic | Haemorrhagic |
| --- | --- | --- | --- | --- |
|  | **OR (95% CI)** | **OR (95% CI)** | **OR (95% CI)** | **OR (95% CI)** |
| Overall | 1.01 (0.96-1.05) p=0.81 | 0.98 (0.91-1.04) p=0.49 | 1.01 (0.94-1.09) p=0.73 | 0.91 (0.80-1.04) p=0.19 |
| Women | 0.99 (0.92-1.07) p=0.85 | 0.94 (0.85-1.04) p=0.24 | 1.00 (0.88-1.12) p=0.95 | 0.85 (0.70-1.03) p=0.09 |
| Men | 1.01 (0.96-1.08) p=0.64 | 1.00 (0.92-1.09) p=0.95 | 1.02 (0.93-1.12) p=0.64 | 0.99 (0.82-1.19) p=0.88 |
| Stratifying on 25(OH)D: |  |  |  |  |
| Stratum 1 | 1.10 (0.75, 1.63) | 1.02 (0.56, 1.86) | 0.89 (0.47, 1.67) | 1.73 (0.49, 6.17) |
| Stratum 2 | 1.24 (0.90, 1.73) | 1.45 (0.90, 2.35) | 1.29 (0.77, 2.17) | 1.93 (0.72, 5.21) |
| Stratum 3 | 1.06 (0.81, 1.39) | 1.05 (0.71, 1.55) | 1.08 (0.70, 1.66) | 1.07 (0.48, 2.37) |
| Stratum 4 | 1.04 (0.82, 1.33) | 0.90 (0.65, 1.25) | 0.94 (0.65, 1.34) | 0.76 (0.39, 1.50) |
| Stratum 5 | 0.94 (0.77, 1.14) | 0.99 (0.75, 1.32) | 1.07 (0.79, 1.47) | 0.70 (0.38, 1.27) |
| Stratum 6 | 0.92 (0.77, 1.09) | 0.93 (0.73, 1.20) | 0.97 (0.73, 1.28) | 1.03 (0.62, 1.70) |
| Stratum 7 | 0.91 (0.79, 1.06) | 0.85 (0.69, 1.04) | 0.91 (0.72, 1.15) | 0.81 (0.54, 1.22) |
| Stratum 8 | 0.95 (0.84, 1.08) | 0.91 (0.76, 1.09) | 0.99 (0.80, 1.21) | 0.83 (0.58, 1.18) |
| Stratum 9 | 1.02 (0.91, 1.14) | 1.01 (0.86, 1.17) | 1.06 (0.89, 1.27) | 0.81 (0.59, 1.10) |
| Stratum 10 | 1.07 (0.97, 1.17) | 1.01 (0.88, 1.15) | 1.04 (0.89, 1.20) | 0.92 (0.72, 1.19) |

**Supplementary Table 11:** Mendelian randomization estimates for coronary heart disease in UK Biobank divided into overall (22,363 events) and incident-only CHD (excluding those with prevalent CHD at baseline, 5447 events): odds ratios (95% confidence intervals) per 10 nmol/L higher genetically-predicted concentration of 25(OH)D.

| CHD | Overall | Incident only |
| --- | --- | --- |
|  | **OR (95% CI)** | **OR (95% CI)** |
| Overall | 0.98 (0.95-1.01) p=0.25 | 1.00 (0.94-1.07) p=0.98 |
| Women | 0.98 (0.92-1.04) p=0.54 | 1.01 (0.89-1.15) p=0.85 |
| Men | 0.98 (0.94-1.02) p=0.34 | 1.00 (0.93-1.07) p=0.92 |
| Stratifying on 25(OH)D: |  |  |
| Stratum 1 (lowest) | 1.32 (0.98, 1.77) | 1.37 (0.79, 2.39) |
| Stratum 2 | 0.94 (0.74, 1.19) | 1.03 (0.66, 1.62) |
| Stratum 3 | 0.98 (0.80, 1.20) | 1.16 (0.79, 1.70) |
| Stratum 4 | 1.02 (0.86, 1.22) | 1.11 (0.80, 1.54) |
| Stratum 5 | 0.93 (0.81, 1.07) | 0.94 (0.72, 1.24) |
| Stratum 6 | 0.96 (0.85, 1.09) | 0.95 (0.76, 1.20) |
| Stratum 7 | 0.97 (0.86, 1.08) | 0.94 (0.77, 1.16) |
| Stratum 8 | 0.95 (0.87, 1.04) | 0.92 (0.77, 1.10) |
| Stratum 9 | 0.97 (0.89, 1.05) | 1.00 (0.85, 1.17) |
| Stratum 10 (highest) | 1.00 (0.94, 1.07) | 1.00 (0.87, 1.15) |

**Supplementary Table 12:** Mendelian randomization estimates for main outcomes in UK Biobank in overall population and divided into clinical strata by residual concentration of 25(OH)D for pleiotropic genome-wide score.

| **Study and stratum** | **Mean 25(OH)D (nmol/L)** | **Coronary heart disease** | **Stroke** | **All cause mortality** |
| --- | --- | --- | --- | --- |
| *UK Biobank* |  |  |  |  |
| Overall |  | 0.92 (0.89, 0.95) | 0.96 (0.92, 1.01) | 0.99 (0.96, 1.03) |
| Stratum 1 | 28.7 | 0.96 (0.78, 1.19) | 0.81 (0.61, 1.07) | 0.98 (0.81, 1.19) |
| Stratum 2 | 35.8 | 1.04 (0.86, 1.25) | 1.07 (0.83, 1.38) | 1.12 (0.93, 1.36) |
| Stratum 3 | 41.1 | 0.93 (0.78, 1.11) | 1.01 (0.81, 1.27) | 0.94 (0.79, 1.11) |
| Stratum 4 | 45.9 | 0.82 (0.72, 0.95) | 1.00 (0.81, 1.24) | 0.92 (0.79, 1.07) |
| Stratum 5 | 50.6 | 0.86 (0.76, 0.97) | 0.96 (0.80, 1.15) | 1.02 (0.89, 1.16) |
| Stratum 6 | 55.4 | 0.92 (0.83, 1.03) | 0.87 (0.73, 1.02) | 1.01 (0.89, 1.15) |
| Stratum 7 | 60.5 | 0.93 (0.84, 1.04) | 0.99 (0.85, 1.16) | 0.99 (0.88, 1.12) |
| Stratum 8 | 66.3 | 0.86 (0.78, 0.95) | 0.99 (0.86, 1.14) | 1.03 (0.93, 1.14) |
| Stratum 9 | 73.8 | 0.97 (0.89, 1.05) | 1.01 (0.89, 1.15) | 0.96 (0.88, 1.05) |
| Stratum 10 | 86.5 | 0.95 (0.88, 1.02) | 0.94 (0.85, 1.04) | 0.99 (0.92, 1.07) |

Mendelian randomization estimates using the genome-wide score cannot reliably be attributed to 25(OH)D levels due to pleiotropic associations of the genome-wide score with LDL-cholesterol and triglycerides. These estimates should therefore not be considered as reliable Mendelian randomization estimates.

**Supplementary Table 13:** Study-specific Mendelian randomization estimates for cause-specific mortality for overall population and divided into clinical strata by residual concentration of 25(OH)D.

| **Study and stratum** | **All-cause mortality** | **Cardiovascular mortality** | **Cancer mortality** | **Non-cancer non-cardiovascular mortality** |
| --- | --- | --- | --- | --- |
| *UK Biobank* |  |  |  |  |
| Overall | 1.00 (0.96, 1.03) | 0.99 (0.92, 1.07) | 0.99 (0.94, 1.03) | 1.02 (0.95, 1.09) |
| Stratum 1 | 1.06 (0.81, 1.39) | 1.30 (0.77, 2.22) | 0.85 (0.58, 1.25) | 1.23 (0.78, 1.93) |
| Stratum 2 | 1.06 (0.84, 1.34) | 1.33 (0.80, 2.20) | 0.90 (0.66, 1.24) | 1.16 (0.75, 1.79) |
| Stratum 3 | 1.09 (0.89, 1.33) | 1.22 (0.79, 1.89) | 1.12 (0.86, 1.48) | 0.90 (0.61, 1.31) |
| Stratum 4 | 1.12 (0.94, 1.34) | 1.07 (0.74, 1.54) | 1.15 (0.91, 1.46) | 1.08 (0.77, 1.52) |
| Stratum 5 | 0.95 (0.82, 1.10) | 0.93 (0.67, 1.27) | 0.94 (0.77, 1.15) | 1.00 (0.74, 1.34) |
| Stratum 6 | 0.98 (0.86, 1.11) | 0.91 (0.69, 1.21) | 1.00 (0.84, 1.18) | 1.03 (0.79, 1.34) |
| Stratum 7 | 0.93 (0.83, 1.04) | 0.97 (0.75, 1.26) | 0.91 (0.79, 1.06) | 0.97 (0.77, 1.21) |
| Stratum 8 | 0.97 (0.88, 1.07) | 0.85 (0.68, 1.05) | 1.03 (0.91, 1.18) | 0.95 (0.78, 1.16) |
| Stratum 9 | 0.97 (0.89, 1.05) | 0.94 (0.78, 1.14) | 0.97 (0.87, 1.08) | 0.99 (0.84, 1.18) |
| Stratum 10 | 1.01 (0.94, 1.08) | 0.95 (0.82, 1.11) | 1.01 (0.92, 1.10) | 1.06 (0.92, 1.22) |
| *Copenhagen studies* |  |  |  |  |
| Overall | 0.89 (0.80, 0.99) | 1.12 (0.95, 1.31) | 0.88 (0.77, 1.01) | 0.93 (0.81, 1.06) |
| Stratum 1 | 0.95 (0.66, 1.37) | 1.04 (0.65, 1.65) | 1.06 (0.68, 1.66) | 0.96 (0.65, 1.41) |
| Stratum 2 | 0.92 (0.60, 1.41) | 0.92 (0.52, 1.63) | 0.90 (0.53, 1.53) | 0.98 (0.61, 1.59) |
| Stratum 3 | 0.91 (0.60, 1.38) | 1.05 (0.57, 1.90) | 0.87 (0.51, 1.48) | 0.88 (0.52, 1.49) |
| Stratum 4 | 0.93 (0.61, 1.42) | 1.13 (0.61, 2.10) | 0.90 (0.51, 1.59) | 0.87 (0.50, 1.51) |
| Stratum 5 | 0.93 (0.60, 1.44) | 1.34 (0.70, 2.57) | 0.82 (0.48, 1.39) | 0.89 (0.51, 1.55) |
| Stratum 6 | 0.90 (0.57, 1.43) | 1.34 (0.67, 2.68) | 0.84 (0.47, 1.52) | 0.89 (0.50, 1.61) |
| Stratum 7 | 0.88 (0.57, 1.38) | 1.31 (0.66, 2.63) | 0.85 (0.46, 1.55) | 0.92 (0.52, 1.65) |
| Stratum 8 | 0.81 (0.51, 1.28) | 1.17 (0.56, 2.47) | 0.79 (0.42, 1.45) | 0.98 (0.53, 1.80) |
| Stratum 9 | 0.81 (0.52, 1.27) | 1.10 (0.54, 2.25) | 0.84 (0.45, 1.55) | 0.92 (0.50, 1.71) |
| Stratum 10 | 0.83 (0.56, 1.23) | 0.99 (0.49, 1.98) | 0.95 (0.54, 1.68) | 0.99 (0.58, 1.69) |

Estimates (95% confidence intervals) represent odds ratio per 10 nmol/L higher genetically-predicted concentration of 25(OH)D.

**Supplementary Figure 1:** Mean and spread of 25(OH)D measurements divided by data source and assay type. Solid error bars represent 95% confidence intervals (CI) for the mean, dashed error bars represent +/- 1 standard deviation (SD).

Assay abbreviations: RIA, Radioimmunoassay; IMA, Immunometric assay; CPB, Competitive protein binding; HPLC-MS, High-performance liquid chromatography mass spectrometry; ECLIA, Electro-chemiluminescence immunoassay; CLIA, Chemiluminescence immunoassay.

**Supplementary Figure 2:** Associations of the focused (primary) genetic risk score for 25(OH)D concentrations with cardiovascular traits in UK Biobank

Estimates for all continuous traits expressed in standard deviation units. Estimates for the binary traits (smoking and Type 2 diabetes status) are log odds ratios. Associations are scaled to a 10 nmol/L increase in genetically-predicted 25(OH)D concentrations (10 nmol/L = 0.51 standard deviations).

Associations were estimated in UK Biobank with adjustment for age at baseline, sex, centre, and 10 genomic principal components.

The association with body mass index represents a 0.017 kg/m^2^ increase per 1 standard deviation increase in the GRS (p = 0.035). The association with HDL-cholesterol represents a 0.002 mmol/L increase per 1 standard deviation increase in the GRS (p = 0.001).

**Supplementary Figure 3:** Associations of the genome-wide score with cardiovascular traits in UK Biobank

Estimates for all continuous traits expressed in standard deviation units. Estimates for the binary traits (smoking and Type 2 diabetes status) are log odds ratios. Associations are scaled to a 10 nmol/L increase in genetically-predicted 25(OH)D concentrations (10 nmol/L = 0.51 standard deviations).

Associations were estimated in UK Biobank with adjustment for age at baseline, sex, centre, and 10 genomic principal components.

The association with LDL-cholesterol represents a 0.024 mmol/L (0.94 mg/dL) decrease in LDL-cholesterol per 1 standard deviation increase in the GRS (p = 1×10^-72^). The association with triglycerides represents a 0.032 mmol/L (2.83 mg/dL) decrease in triglycerides per 1 standard deviation increase in the GRS (p=3×10^-80^). A one standard deviation increase in the GRS corresponds to a 4.2 nmol/L increase in 25(OH)D concentrations.

As the genome-wide score is strongly associated with LDL-cholesterol and triglycerides, and as these are strong risk factors for cardiovascular disease and mortality, Mendelian randomization estimates based on this choice of variants are not reliable.

**Supplementary Figure 4:** Observational associations of 25(OH)D concentrations with outcomes (age and sex adjusted only).

Reference value is 50 nmol/L. The shaded area represents the 95% confidence interval for the dose—response curve. Study-specific analyses involved fractional polynomial modelling of continuous associations of 25(OH)D and outcomes using Cox regression stratified by sex, and (where appropriate) trial arm and centre, and adjusted for age at blood draw for 25(OH)D measurement, followed by random effects meta-analysis (see **Methods**).

**Supplementary Figure 5:** Study-specific dose**—**response curves for associations of 25(OH)D concentrations with outcomes combined across data sources (top) and separated by data source (bottom) adjusted for conventional cardiovascular risk factors.

Reference value is 50 nmol/L. The shaded area represents the 95% confidence interval for the dose—response curve. Study-specific analyses involved fractional polynomial modelling of continuous associations of 25(OH)D and outcomes using Cox regression stratified by sex and (where applicable) centre or trial arm, and adjusted for conventional cardiovascular risk factors, namely: age at blood draw for 25(OH)D measurement, calendar month of blood draw, smoking status (current versus other), total cholesterol, high-density lipoprotein (HDL) cholesterol, systolic blood pressure, known history of diabetes, and body mass index, followed by random effects meta-analysis (**see Methods**).

The EPIC-CVD study was specifically designed as a case-cohort study of CVD outcomes therefore does not contribute to analysis of non-CVD outcomes nor all-cause mortality.

**Supplementary Figure 6:** Dose**—**response association of 25(OH)D concentrations with outcomes by deciles (top) and categories (bottom).

Reference value is decile 5 (close to 50 nmol/L) in top figure and is the adequate clinical subgroup (>75 nmol/L) in the bottom figure. The associations of 25(OH)D and outcomes were modelled using Cox regression stratified by sex and (where applicable) centre or trial arm, and adjusted for conventional cardiovascular risk factors, namely: age at blood draw for 25(OH)D measurement, calendar month of blood draw, smoking status (current versus other), total cholesterol, high-density lipoprotein (HDL) cholesterol, systolic blood pressure, known history of diabetes, and body mass index, followed by random effects meta-analysis (**see Methods**).

**Supplementary Figure 7:** Associations between the genetic risk score and 25(OH)D concentrations in strata of 25(OH)D concentrations in: a) UK Biobank, b) Copenhagen studies, and c) EPIC-CVD.

a.
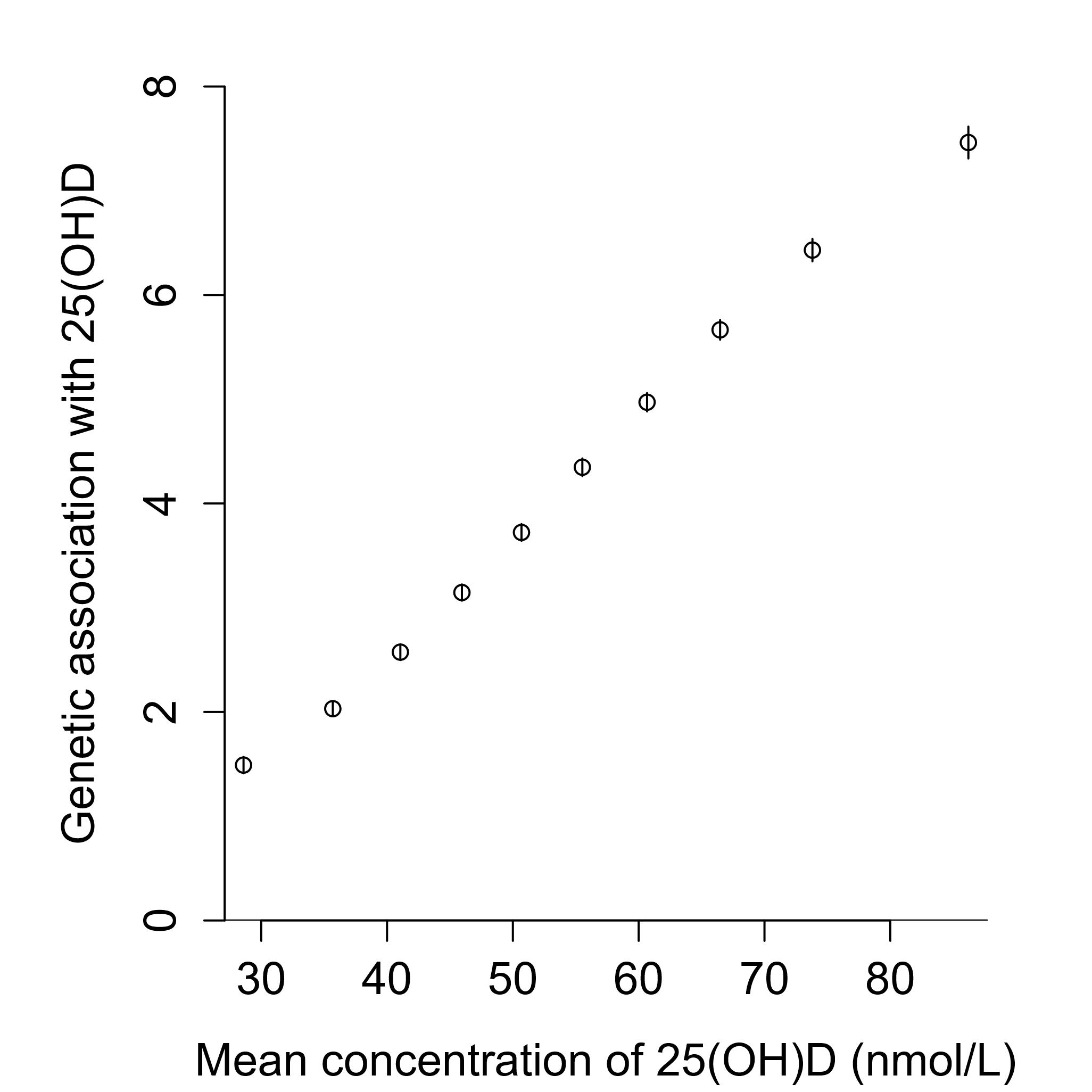
 b.
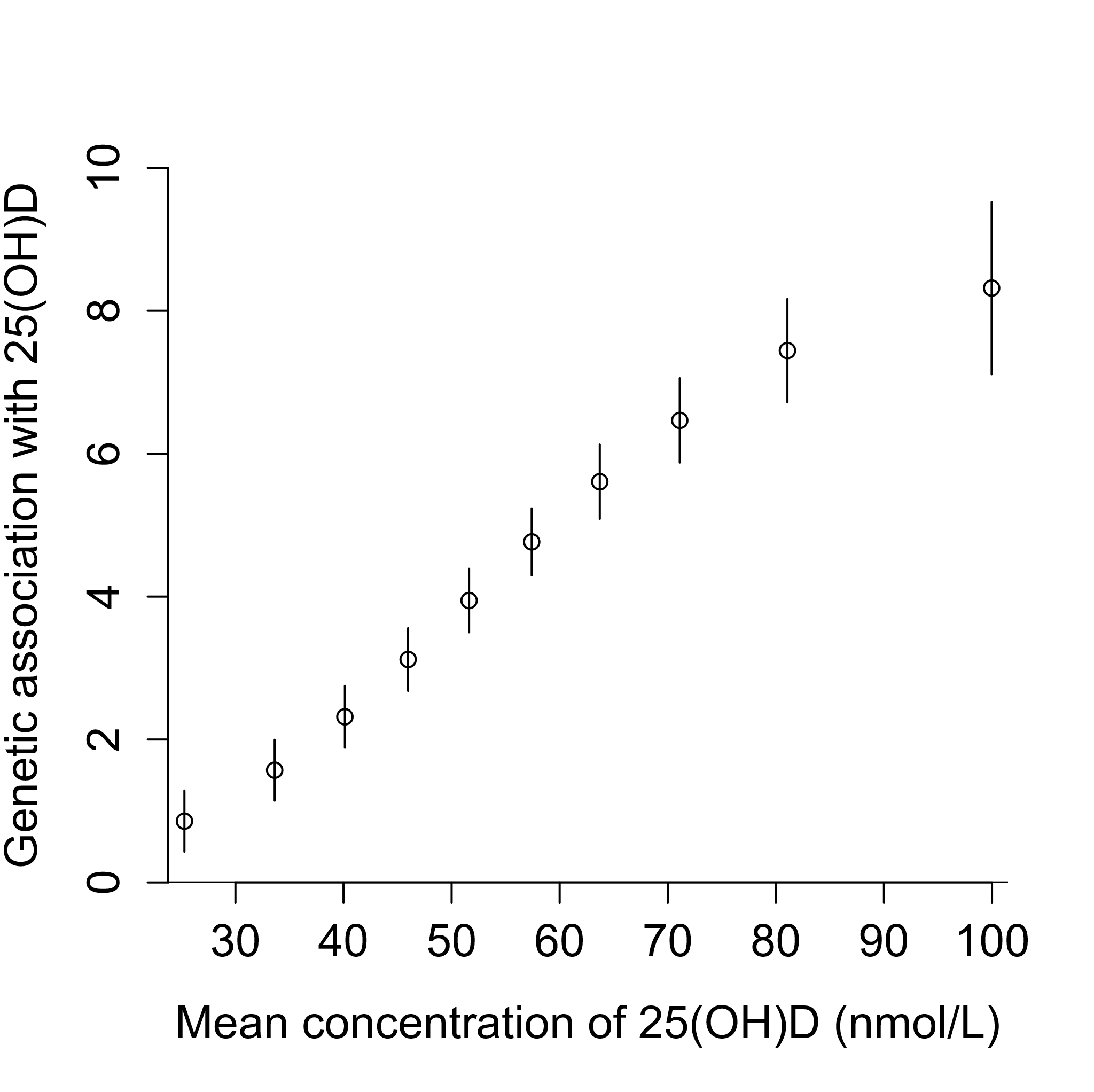


c.
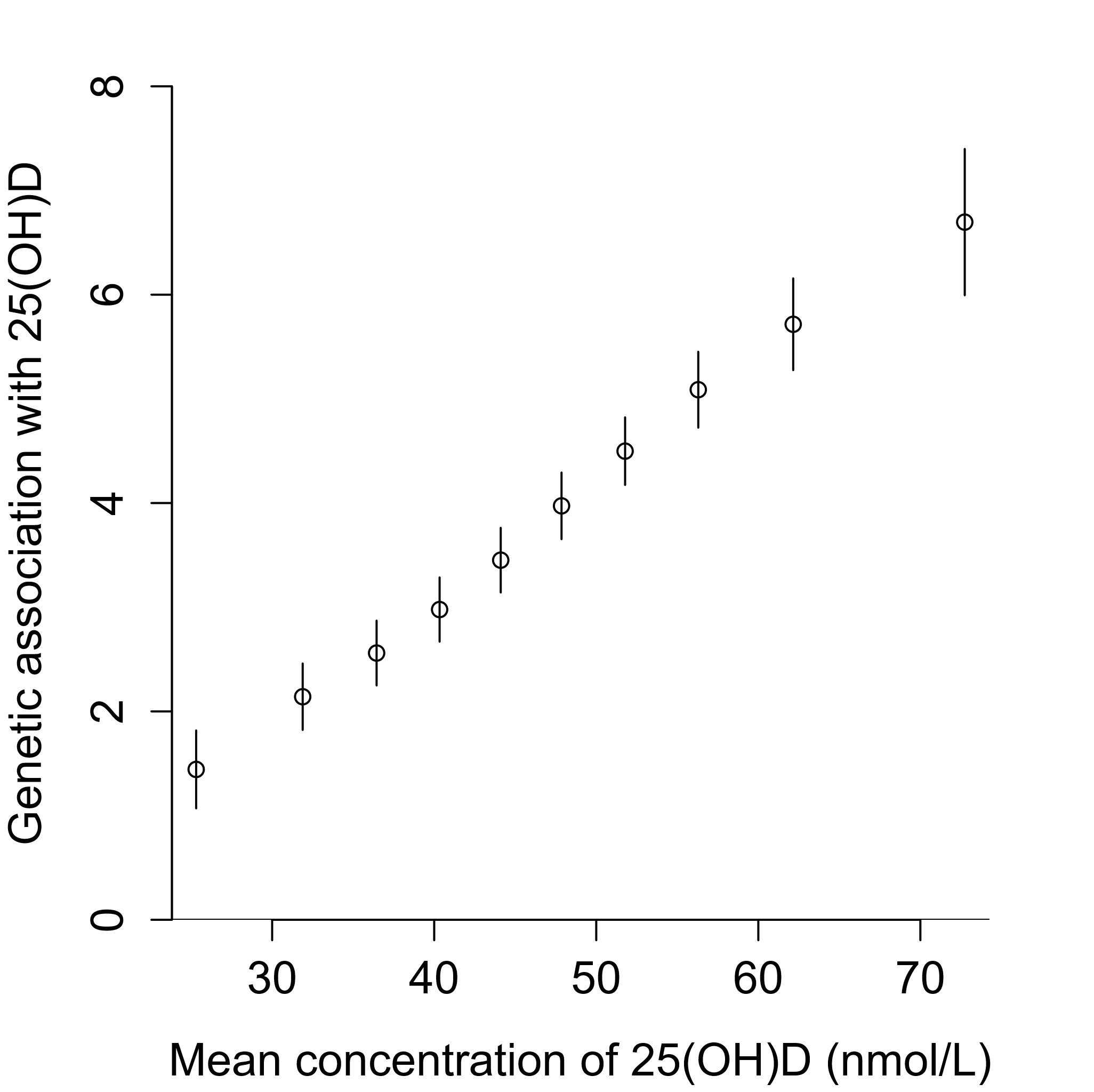


Estimates (95% confidence intervals) represent the association with 25(OH)D in nmol/L per 1 standard deviation increase in the genetic risk score (GRS). For UK Biobank, the genetic association with 25(OH)D in the lowest decile is 1.50 nmol/L; the genetic association with 25(OH)D in the highest decile is 7.51 nmol/L. The ratio between these values is 5.0. Corresponding values are 0.89 nmol/L and 8.59 nmol/L (ratio 9.7) in the Copenhagen studies; and 1.46 nmol/L and 6.74 nmol/L (ratio 4.6) in EPIC-CVD.

**Supplementary Figure 8:** Stratified Mendelian randomization estimates for mortality outcomes in UK Biobank from residual and doubly-ranked methods.


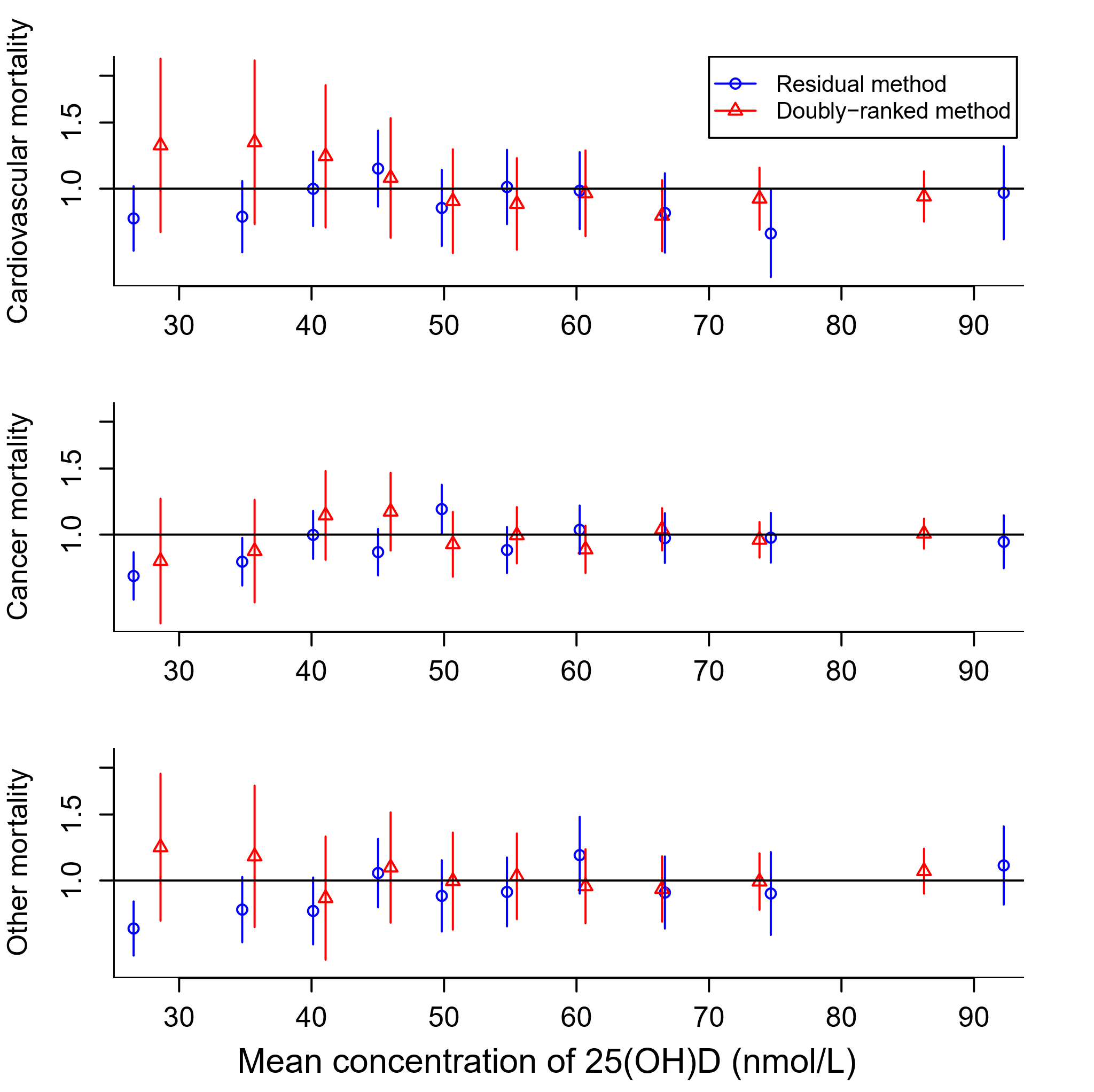


Estimates (95% confidence intervals) represent odds ratios per 10 nmol/L higher genetically-predicted concentration of 25(OH)D in strata of the population defined by residual concentration of 25(OH)D. Estimates from the residual method (not reliable when the genetic effect on the exposure vary in the population) are shown as blue triangles. Estimates from the doubly-ranked method (more reliable when the genetic effect on the exposure vary in the population) are shown as red triangles.

**List of Emerging Risk Factors Collaboration / Vitamin D Studies Collaboration Investigators**

Deutsche Diabetes Dialyse Studie (4D): Christiane Drechsler;

Auckland Calcium Study (AUCKLAND): Mark J Bolland, Ian Reid;

Bruneck Study (BRUN): Johann Willeit, Georg Schett, Peter Santer;

British Women's Heart and Health Study (BWHHS): Reecha Sofat, Julie Taylor, Caroline Dale;

Calcium Intake Fracture Outcome Study (CAIFOS): Richard L Prince;

Caerphilly Prospective Study (CAPS): Yoav Ben-Shlomo, John Gallacher;

Copenhagen City Heart Study (CCHS): Gorm B Jensen, Ruth Frikke-Schmidt, Stig Egil Bojesen;

Copenhagen General Population Study (CGPS): Marianne Benn, Anders B Wulff, Signe V Krogh;

Danish Osteoporosis Prevention Study (DOPS): Louise Lind Schierbeck;

European Prospective Investigation of Cancer Bone Mineral Density (EPICBMD): Stephen Kaptoge;

European Prospective Investigation of Cancer Norfolk Study (EPICNOR): Nicholas Wareham;

Epidemiologische Studie zu Chancen der Verhütung und optimierten Therapie chronischer Erkrankungen in der älteren Bevölkerung (ESTHER): Ben Schöttker, Anna Zhu, Bernd Holleczek;

Hertfordshire Cohort Study (HCS): Elaine Dennison, Karen Jameson;

Herz-und Diabeteszentrum Nordrhein-Westfalen (HDZNRW): Stefanie Schulze Schleithoff, Sabine Frisch;

The Inter99 cohort (INTER99): Allan Linneberg, Tea Skaaby, Line Lund Kårhus;

Longitudinal Aging Study Amsterdam (LASA): Renate T de Jongh, Marjolein Visser;

Ludwigshafen Risk and Cardiovascular Health (LURIC): Harald Dobnig;

Multi-Ethnic Study of Atherosclerosis (MESA): Cassianne Robinson-Cohen, David S Siscovick, Bryan R Kestenbaum

The MIDSPAN studies (MIDSPAN): Alex McConnachie, Naveed Sattar, David Morrison

Mini-Finland Health Survey (MINIFIN): Annamari Lundqvist;

Danish Monitoring Trends and Determinants of Cardiovascular Disease 10 year follow-up (MONICA10): Allan Linneberg, Tea Skaaby;

Osteoporotic Fractures in Men (MROS): Peggy M Cawthon;

Third National Health and Nutrition Examination Survey (NHANESIII): Juan R Albertorio;

Prospective Study of Pravastatin in the Elderly at Risk (PROPSER): J Wouter Jukema, Stella Trompet, Patricia Kearney;

Study of Health in Pomerania-1 (SHIP-1): Marcus Dörr, Henry Völzke, Matthias Nauck;

Study of Osteoporotic Fractures-1 (SOF1): Peggy M Cawthon;

Study of Osteoporotic Fractures-4 (SOF4): Peggy M Cawthon;

Steno Diabetes Center (STENO): Peter Rossing, Frederik Persson;

Turku-Finland study (TURKUFIN): Jukka Marniemi;

TwinsUK (TWINSUK): Victoria Vazquez;

Uppsala Longitudinal Study of Adult Men (ULSAM): Johan Sundström, Ulf Risérus, Karl Michaëlsson;

Whitehall I Study (WHITEI): Jonathan Emberson, David Leon, Mika Kivimäki.
